# A Bayesian Approach to Extracting Kinetic Information from Artificial Enzymatic Networks

**DOI:** 10.1021/acs.analchem.2c00659

**Published:** 2022-05-12

**Authors:** Mathieu G. Baltussen, Jeroen van de Wiel, Cristina Lía Fernández Regueiro, Miglė Jakštaitė, Wilhelm T. S. Huck

**Affiliations:** Institute for Molecules and Materials, Radboud University Nijmegen, 6525 AJ, Nijmegen, The Netherlands

## Abstract

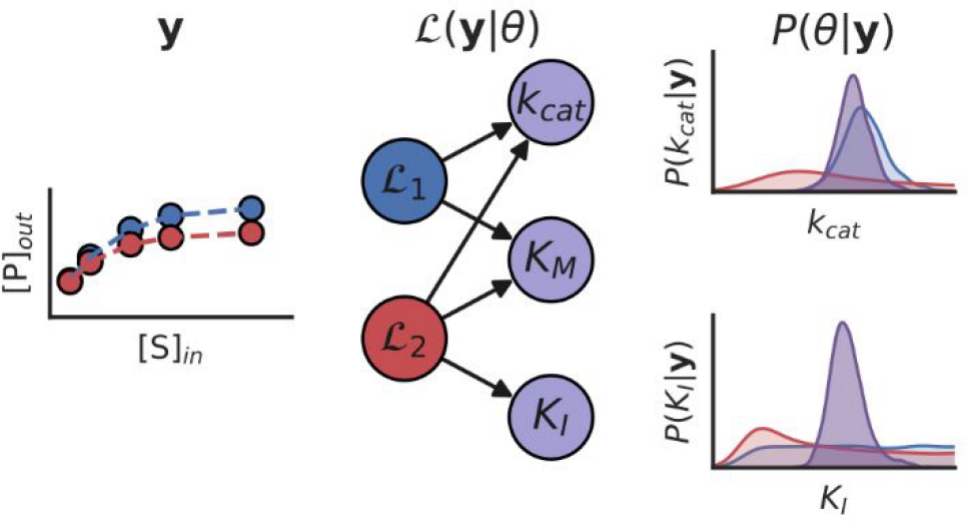

In order to create artificial enzymatic networks capable of increasingly complex behavior, an improved methodology in understanding and controlling the kinetics of these networks is needed. Here, we introduce a Bayesian analysis method allowing for the accurate inference of enzyme kinetic parameters and determination of most likely reaction mechanisms, by combining data from different experiments and network topologies in a single probabilistic analysis framework. This Bayesian approach explicitly allows us to continuously improve our parameter estimates and behavior predictions by iteratively adding new data to our models, while automatically taking into account uncertainties introduced by the experimental setups or the chemical processes in general. We demonstrate the potential of this approach by characterizing systems of enzymes compartmentalized in beads inside flow reactors. The methods we introduce here provide a new approach to the design of increasingly complex artificial enzymatic networks, making the design of such networks more efficient, and robust against the accumulation of experimental errors.

## Introduction

Enzymatic reaction networks (ERNs) play key roles in many cellular processes, such as energy metabolism, signaling pathways, and cell division.^1−3^ The fields of synthetic biology and systems chemistry aim to understand and reproduce the behavior of these ERNs in artificial systems.^4−8^ Previous work has shown the development of small network motifs^9^ by autocatalysis and delayed inhibition,^10^ photochemical control of oscillations by reversible photoinhibitors,^11^ coupling to DNA-based circuits,^12^ logic-gate responses,^13^ pattern-formation,^14^ adaptive responses to environmental perturbations,^15^ and coupling to dynamic environments.^16^ While these networks can show complex behavior, such as oscillations and adaptation, scaling up their size toward metabolic scales remains a significant challenge. To construct complex, yet functional ERNs, estimating the mechanisms and kinetics of the enzymatic reactions in these systems is essential in order to reliably predict the relevant experimental regimes in which a desired functional output will be observed.^17^ But while the development of artificial ERNs with more complex behavior continues, methods are missing to not only obtain realistic kinetic parameter estimate but also simultaneously allow for the evaluation of the relevance and correctness of existing kinetic models.

This lack of accurate and experimentally realistic parameter and mechanism estimation greatly limits the efficient exploration of more complex systems. Furthermore, while the fitting of a model to experimental data is in principle relatively simple, in practice numerous sources of uncertainty are encountered, including experimental errors and unknown inhibitory or allosteric effects. Typically, the kinetic parameters of an enzymatic reaction are estimated from a single data set, using least-squares regression or similar maximum likelihood estimation methods. Although this approach is well-established, there are multiple downsides.^18^ First, sources of uncertainty must be explicitly modeled in, which would require an exact knowledge of the influence of these uncertainties on the final experimental results.^19,20^ Second, this approach often neglects additional sources of data, either from previous or additional experiments or from literature. And last, estimation of enzyme kinetics is often done using rather limited data sets, which should increase the uncertainty of the obtained parameter values, but in practice potentially leads to overfitting of the proposed model.^21,22^

Here, we demonstrate an analysis framework based on Bayesian methods for the inference of kinetic parameters and comparison of reaction mechanisms of compartmentalized enzymes in a flow reactor. Bayesian methods are probabilistic in nature, so that any knowledge of kinetic parameters or reaction mechanisms obtained from experimental data is expressed in terms of probability distributions, instead of specific values. Furthermore, they allow for the explicit incorporation of any information previously obtained on the system in question through the prior, from either literature or previous experiments, resulting in a coherent framework for combining data from different sources.^23^ Additionally, they are ideally suited for estimations that contain uncertainty and a lack of data.^24^ Bayesian methods have been more widely implemented in recent years, mainly due to an increase in available computational power and an increase in general availability of powerful, yet accessible, algorithms. They are used in a wide range of fields, from applications in pure physics,^25^ medicine,^26^ and sociology^27^ to large-scale metabolomics in systems biology^28^ and optimized peak detection in chromatographic methods.^29^ Previous research on the applications of Bayesian methods in enzymatic networks has mostly been attempted from a systems biology perspective, focusing on whole-cell metabolomics,^28,30,31^ or focusing on simulated data sets and evaluating the feasibility of an alternative enzyme rate equation.^32^ The approach introduced here instead focuses on experimental relevance and the combination of multiple data sets, specifically for the construction of encapsulated enzymes in flow, but is readily adoptable in most experimental enzymatic reactions setups, without requiring extensive computational expertise to employ.

## Methods

### Bead Production

Two methods were used to immobilize enzymes through polymerization of the enzyme into polyacrylamide hydrogel beads. The first method consists of enzyme-first functionalization with a 6-acrylaminohexanoic acid succinate linker (AAH-Suc) by coupling to amino groups of lysine using NHS chemistry, followed by the production of hydrogel beads using droplet-based microfluidics by UV-polymerizing water-in-oil droplets containing the functionalized enzyme, acrylamide, *N*,*N*′-methylenebis(acrylamide), and a photoinitiator yielding monodisperse polyacrylamide-enzyme beads (PEBs). In the second method, droplet-based microfluidics techniques are used to produce empty hydrogel beads consisting of acrylamide, *N*,*N*′-methylenebis(acrylamide), acrylic acid, and 2,2′Azobis(2-methylpropionamidine) dihydrochloride. After UV polymerization the carboxyl groups of acrylic acid were activated by EDC/NHS chemistry and later enzyme was coupled via amine groups of lysine residues forming PEBs. Details of the procedure used per type of PEB can be found in the Supporting Information (SI).

### Flow Experiments

Flow experiments were conducted similarly to the description in previous work,^33^ but replacing the inflow of the desired enzymes with the desired volume of PEBs, which remained compartmentalized in a Continuously Stirred Tank Reactor (CSTR) during the experiment. The openings of the reactors were sealed with Whatman Nuclepore polycarbonate membranes (5 μm pore size) to prevent outflow of PEBs. Cetoni Low-Pressure High-Precision Syringe Pumps neMESYS 290N were used to control the dispense of the different solutions, prepared in Gastight Hamilton syringes (2500–10 000 μL), into the CSTR. The precise flow profile of the desired flow rates was programmed using the Cetoni neMESYS software.

To detect and determine outflow concentrations from the CSTR, both online and offline detection was employed. Online absorbance detection was achieved with an Avantes AvaSpec2048 Fiber Optic spectrometer and Avantes AvaLight 355 nm LED combined with a custom designed flow cuvette provided to us by LabM8. Alternatively, offline measurement could be achieved by means of connecting the outflow to a BioRad Model 2110 fraction collector. These fractions could subsequently be probed for NADH absorbance using a Tecan Spark M10 platereader, or probed for ATP, ADP, NAD+, and NADH using a Shidmadzu Nexera X3 HPLC.

Further details on the instrumentation and experimental protocols can be found in the Supporting Information.

### Modeling of Enzyme Kinetics in Flow

We generally assume that the enzymatic reactions behave according to Michaelis–Menten-like mechanisms, although other mechanisms might also be considered, and we add flow-dependent terms to model the dynamics of the flow reactor. Inclusion of these flow terms yields the following system of Ordinary Differential Equations (ODEs) for a single-substrate single-product reaction:
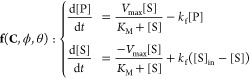
where *V*_max_ = *k*_cat._[*E*] and *K*_M_ are the kinetic parameters ϕ that generally need to be estimated, and where we introduce a set of control parameters θ, in the form of [*S*]_in_, the effective substrate concentration flown into the reactor, and , the flow constant. Measurements of the product concentration are performed when the system has reached steady-state conditions (), resulting in a set of steady-state concentrations [P]_ss_ and [S]_ss_. These values can then be used in a fitting procedure to obtain kinetic parameter estimates.

### Creation of Bayesian Models

In a Bayesian approach, the probability distributions for parameters of interest are obtained by application of Bayes’ theorem
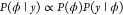
which relates the posterior probability *P*(ϕ|*y*) of a specific parameter value ϕ given the data *y* observed during an experiment, to the likelihood *P*(*y*|ϕ) of observing that specific data given the parameter value, and any previously available information on the parameter, the prior *P*(ϕ). As the likelihood is a function of both the observed data and the values of the kinetic parameters, and not a probability distribution, we write .

In the case of a single-substrate, steady-state enzymatic network, the observed data *y* is given simply by the set of observed steady-state concentrations [P]_ss_ at specific experimental conditions [S]_in_ and *k*_f_, which we here consider to be exactly known, while the parameter ϕ can be any of the kinetic parameters that is unknown, such as *k*_cat._ or *K*_M_. Furthermore, because the data we collect are inherently noisy, we assume that the concentrations we observe are part of a normal distribution [P]_obs_ ≈ *N*([P]_ss_,σ) with a mean equal to the true steady-state concentration and an unknown standard-deviation σ. Depending on the measurement techniques, a different probability distribution that correctly incorporates the physical details of the noise-generating observation model might be more suitable. This assumption allows us to write down the form of the likelihood , where [P]_ss_ = *g*(*ϕ,θ*) is a function of the kinetic parameters and the experimental conditions. The likelihood incorporates most sources of uncertainty, such as the intrinsic fluctuations of product concentration at steady state and noise from the used measurement technique, inside the uncertainty-term σ. This parameter is then inferred simultaneously with the kinetic parameters *k*_cat._ and *K*_M_, allowing us to directly estimate the uncertainty in our observations as well. In addition, if any uncertainties exist in the experimental conditions, these can be incorporated into the analysis as informed priors, and inferred alongside the other parameters. Any other sources of uncertainty, such as inconclusive data, or a wrong assumed reaction-mechanism, are implicitly encoded into the posterior probability distributions of the kinetic parameters.

We use the Python package PyMC3^34^ and custom-written likelihood functions for observed steady-state concentrations, from which we can sample with the No–U-Turn Sampler (NUTS^35^) by inclusion of likelihood gradients. The steady states can be obtained either by symbolically solving f(*C*,*ϕ*,θ) = 0, or by numerical root-finding of the vector-function f. The gradients for the numerical steady states are obtained from the Implicit Function Theorem, which relates the sensitivity of the steady-state concentrations  to the kinetic parameters without needing to explicitly write down an expression for the steady-state concentrations, via *∂g*/*∂ϕ*(*ϕ,θ*) = −[*∂f*/*∂c*(*C*,ϕ,θ) ]^−1^ [*∂f*/*∂ϕ*(*C*,ϕ,θ)], while gradients for symbolic steady-states are automatically obtained via automatic differentiation in PyMC3. Using the NUTS-algorithm, we can obtain correlated probability distributions for the value of every kinetic parameter of interest. Implementations of the computational models used in this paper and the scripts to generate the figures can be found in the Supporting Information in the ‘Model details and sampling diagnostics’ section and on the associated Github page.

## Results and Discussion

### Obtaining Improved Accuracy from Correlated Parameter Estimates

**Figure 1:**
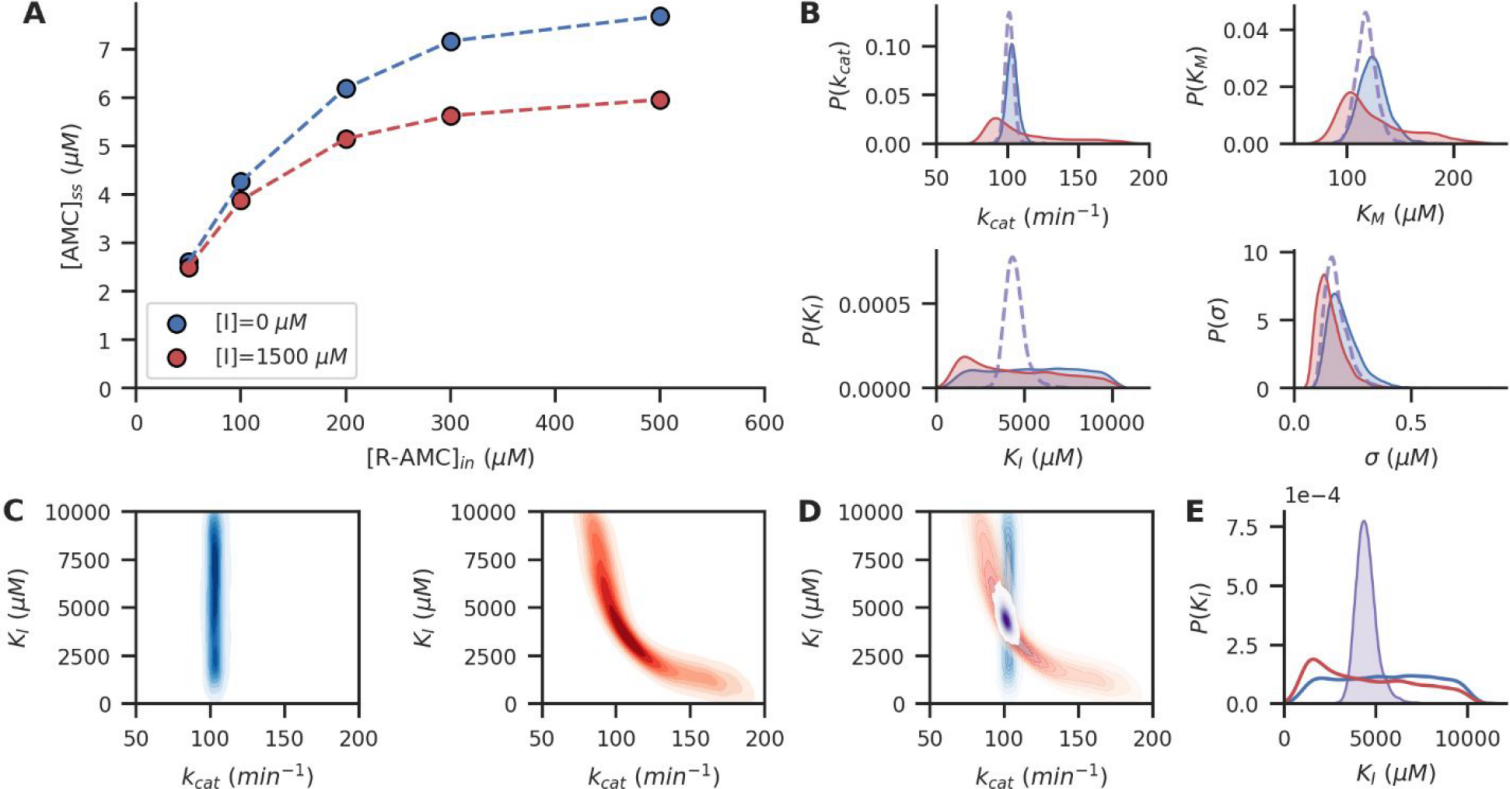
(A) Steady-state concentrations of R-AMC cleavage by Trypsin, with and without inhibitor (AAA-AMC). (B) Posterior parameter estimates obtained from the data without inhibitor present (blue) and with inhibitor present (red). Combining both data sets in one model yields more precise posterior estimates (dashed, purple). (C) Posterior correlation plots of *k*_cat._ and *K*_I_ from the data without inhibitor present (blue, left), showing no correlation, and with inhibitor present (red, right), showing high nonlinear correlation. (D) Combining data from both experiments yields a new posterior distribution (purple) that exactly corresponds to the intersection from the two experiments separately. (E) Comparison of posterior *K*_I_ estimates from the individual data sets (blue, red) to the estimate obtained from the combined data set (purple).

We first show the relevancy of our Bayesian approach by estimating the kinetic parameters of Trypsin PEBs cleaving a substrate (Cbz-Arg-7-amino-4-methylcoumarin, R-AMC) while in the presence of an inhibitor (Suc-Ala-Ala-Ala-7-amino-4-methylcoumarin, AAA-AMC), shown in Figure 1. The mathematical model for this system assumes Michaelis–Menten-type kinetics with an uncompetitive inhibitor effect and can be found in the Supporting Information and corresponding computational notebooks. Two experiments were performed, one where the inhibitor was absent and one where the inhibitor was present (Figure 1A). Both experiments on their own did not yield enough information to obtain conclusive estimates of all kinetic parameters involved (*k*_cat._, *K*_M_, *K*_I_), as shown in Figure 1B. Clearly, from the experiment without inhibitor relatively precise estimates can be obtained on *k*_cat._ and *K*_M_, but no information is obtained on the value of the inhibition constant *K*_I_. Thus, our posterior estimate of the inhibition constant is equivalent to our prior estimate (a uniform distribution between 1 and 10 × 10^3^ μM). In contrast, from the experiment with inhibitor present, a posterior estimate for the inhibition constant can be obtained, albeit not a precise one. Additionally, from this experiment alone, the posterior estimates for the other kinetic parameters are also uncertain.

However, while the posterior estimates of the individual parameters remain uncertain, we do obtain additional information by analyzing the posterior correlations, shown in Figure 1C. While the experiment without inhibitor does not show any correlation between the value of the estimated *k*_cat._ and *K*_I_ values, the experiment with inhibitor present shows a nonlinear correlation between low estimated values of *k*_cat._ and high values of *K*_I_, and vice versa.

Combining data from both experiments in a single likelihood function allows us to combine the certainty of the parameter estimates present in the first experiment with the highly correlated parameter estimates of the second experiment, to obtain a posterior distribution that is essentially an intersection of those obtained from the individual experiments (Figure 1D). As expected, this allows us to obtain a much more precise estimate of the inhibitor constant, as shown in Figure 1E. Moreover, this procedure yields improved estimates for every parameter in the system, not just the inhibition constant, which can be observed in Figure 1B.

Consequently, the Bayesian approach greatly simplifies the iterative addition of experimental data to update parameter estimates. As shown here, subsequent measurements of enzyme activity in the presence of an inhibitor not only will allow an estimation of the inhibition constant but also retro-actively improves the estimates for the Michaelis constant *K*_M_ and the turnover number *k*_cat._.

### Combining Diverse Experimental Data Sets

More complex ERNs introduce a number of additional challenges in modeling the system’s behavior. One of these challenges is combining data from a diverse range of experiments, both with variations in experimental conditions, and variations in network topologies due to the enzymes that are present. Additionally, for some experiments only partial data can be obtained, for example in the case where only substrates involved in a single reaction can be observed, while substrates from a different reaction remain undetected.

**Figure 2:**
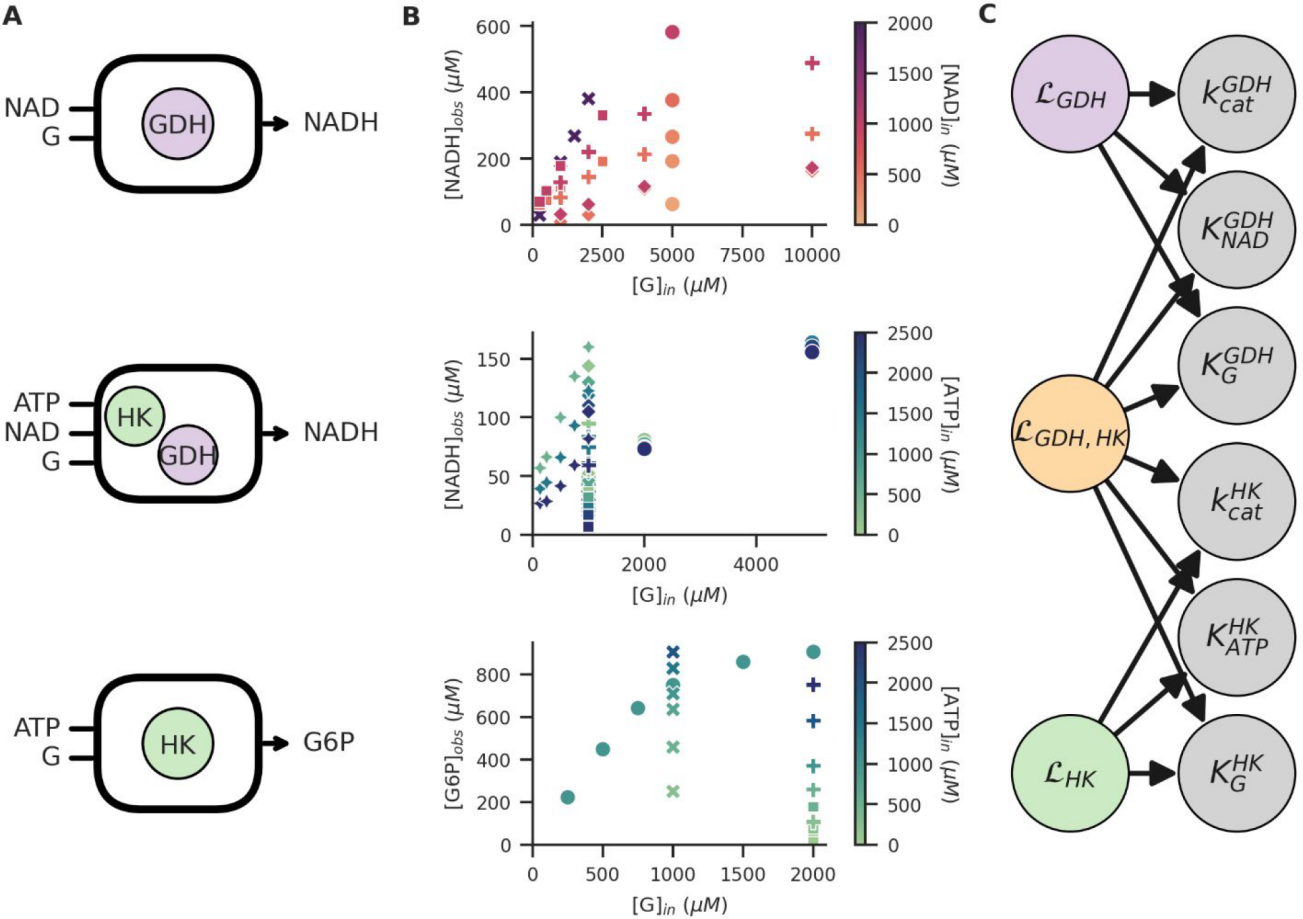
(A) Three different ERN topologies are used in different reactors, and at different experimental conditions (varying input concentrations and volume of PEBs). (B) Plots showing all collected observations at different input concentrations of glucose (*x*-axis) and cofactor (color intensity). Experiment runs for every topology are indicated by symbol. The observed species is topology-dependent. (C) Schematic of the causal network relating the observation likelihoods  to the inferable parameters, where likelihoods corresponding to either the GDH or HK topology only relate to a subset of the parameters. The combined GDH,HK likelihood relates to every kinetic parameter in the probabilistic model.

In Figure 2, we show how data obtained from these different types of experiments can be captured in a single probabilistic model. In Figure 2A, we distinguish between three different network topologies, two with only a single type of enzyme PEB present, either glucose-dehydrogenase (GDH) or hexokinase (HK), and one where both enzymes PEBs are present simultaneously. For all three topologies, multiple experiments are performed at different conditions, such as different substrate input concentrations and PEB volumes used. For the two single-enzyme topologies, detection of a single substrate is enough for full observability of the network (through stoichiometric conservation), while, for the combined GDH+HK topology, only NADH is observed. Thus, the substrates involved in the hexokinase-reaction are not directly detected. The resulting complex data set is shown in Figure 2B. All three topologies have a corresponding likelihood function that relates the observations to the kinetic parameters in question, as shown schematically in Figure 2C (see the SI for the programmatic implementation of these likelihoods). While the GDH+HK system does not allow for full observability of the network, its likelihood does allow us to correlate the GDH and HK kinetic parameters, consequently leading to improved estimates of all parameters involved.

**Figure 3:**
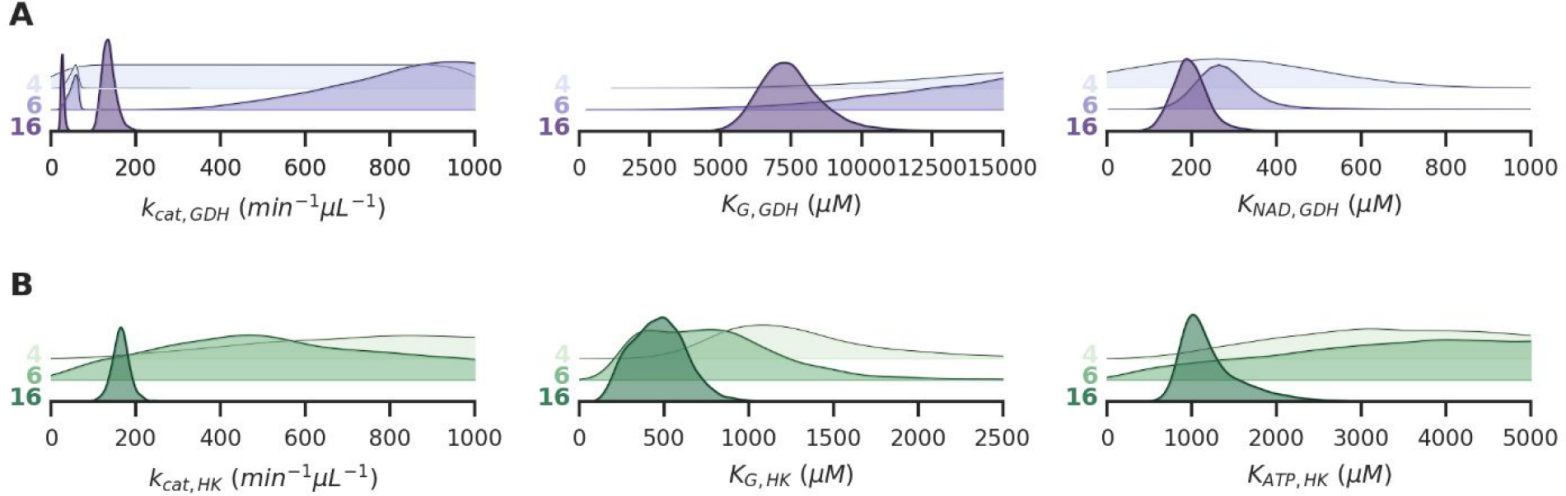
(A,B) Posterior parameter estimates obtained from the model combining all three (GDH, HK, GDH+HK) observation likelihoods. For every parameter, the distributions are shown for 3 different data set sizes, with respectively 4, 6, and 16 experiments included. Distributions are shifted and scaled to increase visibility. For the GDH *k*_cat._, two estimates are obtained because PEBs with two different enzyme concentrations were used in different experiments.

The resulting posterior estimates of combining all available data are shown in Figure 3. For the GDH PEBs, two different batches were used with different enzyme concentration, resulting in two distinct effective *k*_cat._ parameters. Estimation of their respective values are performed under the assumption that the *K*_M_ for the two substrates remain the same for both batches. By directly encoding this assumption into the combined likelihood functions, observations on both batches become relevant for estimation of all the parameters involved.

Correlating the parameter estimates of the individual enzymes through a joint likelihood function allows us to potentially improve parameter estimates by observing a system not directly related to those parameters. Thus, as more observations are made, any parameter estimates will increase in accuracy simply by the inclusion of more data. This iterative improvement of estimates as more data becomes available is shown by the gradual shrinkage of posterior distributions, implying that the estimates become more precise. This improvement is most pronounced when little data are available (for example from 4 to 6 experiments), but gradually becomes less for larger data sets (the same figure extended to stepwise addition of every experiment up to 16 can be found in the SI), and eventually converges to a final posterior distribution. For these final posterior distributions, adding new data will not significantly alter the results, but the estimates will increase in robustness and become less susceptible to outliers in the data.

**Figure 4:**
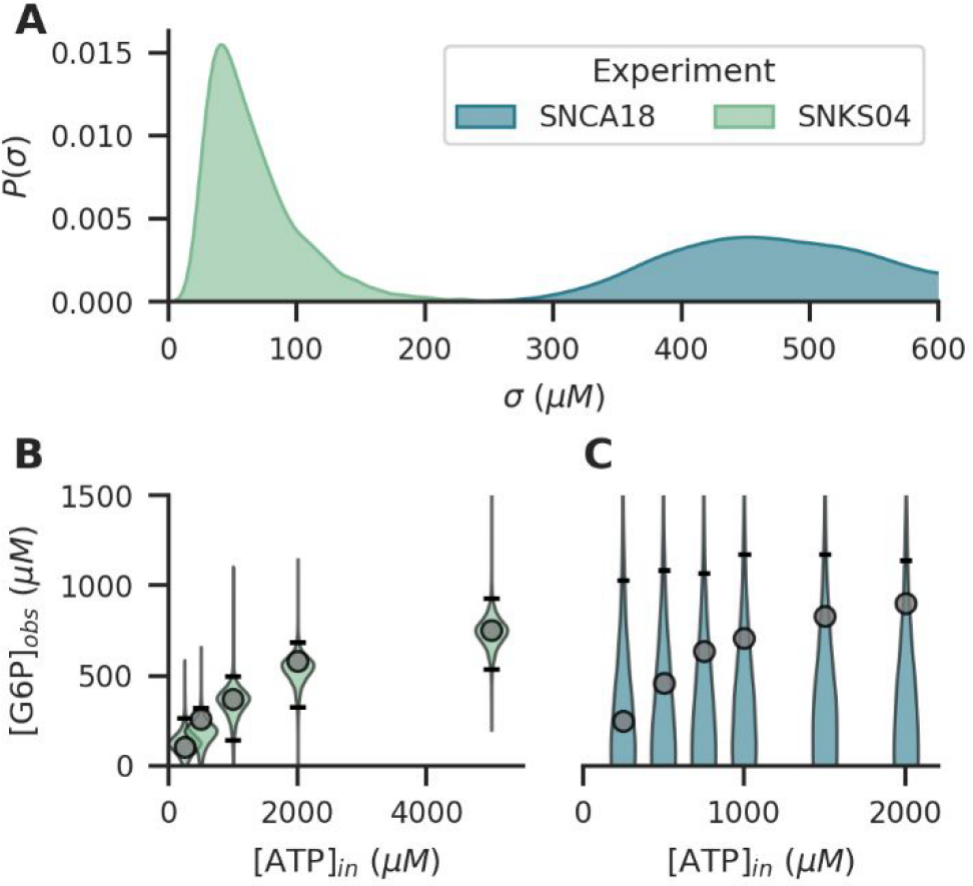
(A) Posterior experimental uncertainty estimates for two specific HK-experiments, obtained from the posterior distributions calculated from the full data set of all experiments. One experiment (green, SNKS04) has a low estimated uncertainty, while the other experiment (blue, SNCA18) has a much higher estimated uncertainty. (B) Associated observed data points of the low-uncertainty experiment, the posterior predictive distribution of expected observations, and 95% CI quantiles (black). (C) Associated observation data points of the high-uncertainty experiment, the posterior predictive distributions of the expected observations, and 95% CI quantiles (black).

Additionally, by estimating the uncertainty in every experiment individually, it becomes more practical for a large number of experiments to determine which ones have corresponding results, and which ones are potential outliers or contain experimental errors. This can be observed especially in the uncertainty estimates for two specific HK experiments, as shown in Figure 4. One experiment, with experiment code SNKS04, has a relatively low uncertainty estimates (Figure 4A) and correspondingly, the posterior predictions obtained from the model are similar to the actual observations (Figure 4B). However, another experiment stands out with a much higher uncertainty estimate (σ ≈ 400–600 μM), which indicates some unknown error in the observations made during that experiment. Consequently, the posterior predictions show a very large spread and do not correlate well with the actual observations (Figure 4C). Importantly, these uncertainty estimates are obtained by conditioning of the individual observations on the complete data set of all experiments. Large uncertainty estimates therefore imply results that do not correspond with most other performed experiments.

By estimating the uncertainty parameters alongside all of the kinetic parameters, individual experiments are allowed to be “wrong”, and consequently influence the final parameter estimates less than other experiments. While not a solution for badly performed experiments, it does protect against drawing incorrect conclusions from incorrect data. Consequently, the uncertainty estimates indirectly act as an automatic weighting factor for individual experiments, where experiments with higher estimated uncertainty are less relevant toward the kinetic parameter estimations. It can also function as a key indicator for experiments influenced by an unknown source of error or systematic bias, especially in cases where large data sets collected over longer time periods are involved.

### Comparing Reaction Mechanism Hypotheses

The microscopic mechanisms underlying enzymatic reactions often allow for the creation of more complex kinetic models than simple Michaelis–Menten kinetics. However, a more complex model, with more kinetic parameters, does not necessarily imply a more useful model. Instead, in the presence of uncertain data, it can lead to overfitting and unrealistically high certainty in the estimates.

**Figure 5:**
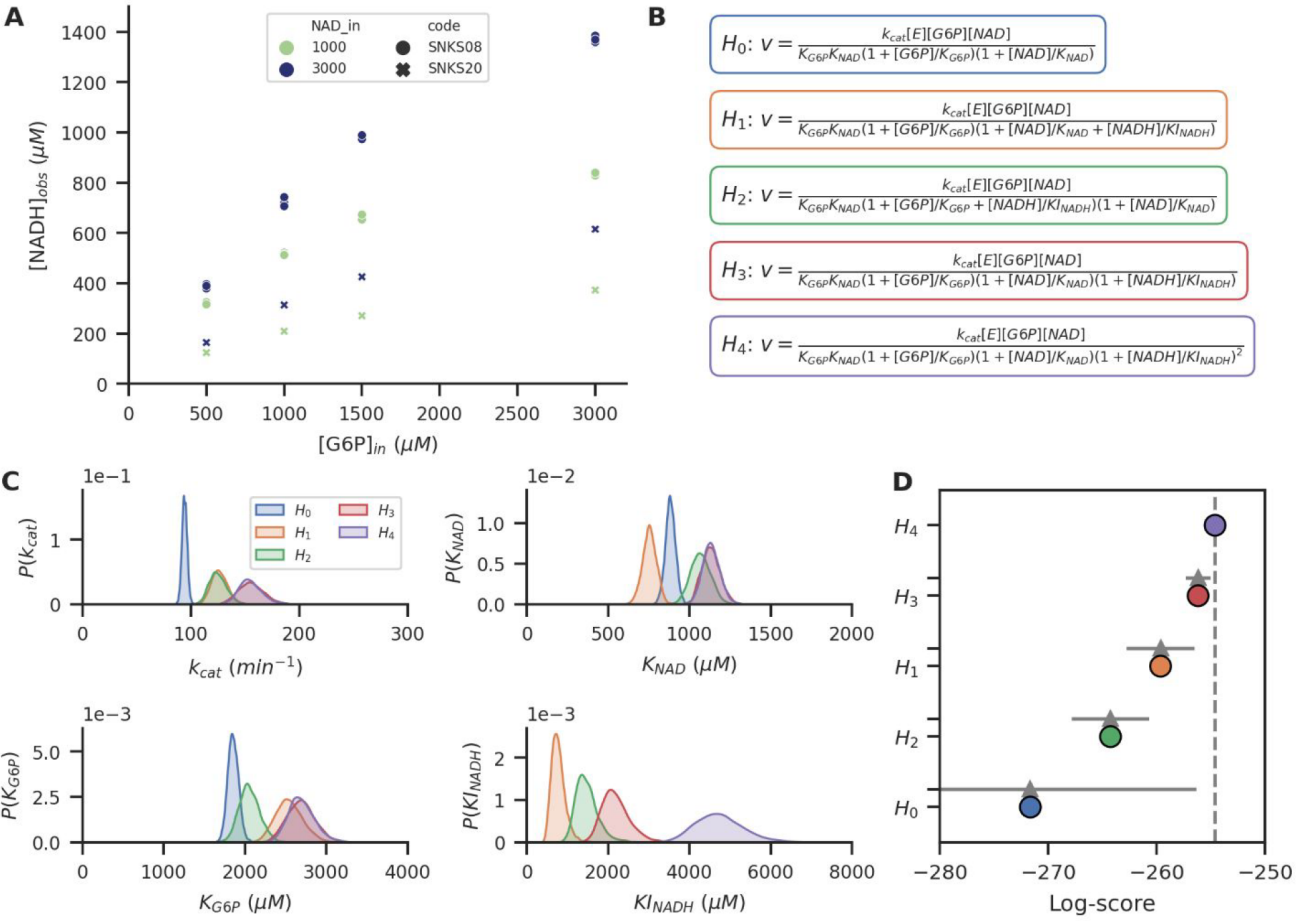
(A) Steady-state concentrations obtained during two experiments, from a G6PDH system at different glucose-6-phosphate input concentrations and NAD input concentrations. Every measurement point is obtained in triplicate. (B) Five different hypotheses for Michaelis–Menten mechanisms without (*H*_0_), and with NADH product inhibition (*H*_1_–*H*_4_). Only the reaction rate is shown, but full sets of ODEs with additional flow terms are used in the probabilistic model. (C) Posterior parameter estimates for all five hypotheses. *H*_0_ does not include an inhibition constant *KI*_NADH_, but all other hypotheses do. (D) Comparison of the leave-one-out cross-validation information criterion for all five hypotheses (colored), and standard errors of the difference in information criterion with respect to the top-ranked model (gray). A higher log-score indicates a hypothesis with more predictive power.

In Figure 5 we show how the posterior estimates obtained using our Bayesian approach can be used to compare different hypotheses for the reaction mechanism and associated kinetics of glucose-6-phosphate dehydrogenase (G6PDH) PEBs. From a set of experiments performed at varying experimental conditions (Figure 5A), we propose a number of different hypotheses describing the suspected mechanism of product inhibition by NADH on the reaction rate (Figure 5B). We also include a 0-hypothesis describing a mechanism where the formation of NADH has no inhibiting effect, although the inclusion of a 0-hypothesis is not necessary for using this methodology.

We consider four modes of NADH inhibition: competitive inhibition of the NAD-binding site (*H*_1_), competitive inhibition of the G6P-binding site (*H*_2_), noncompetitive inhibition of the enzyme activity (*H*_3_), and cooperative noncompetitive inhibition of the enzyme activity (*H*_4_). All five hypotheses result in posterior distributions that give well-defined parameter estimates (Figure 5C), from which it is difficult to conclude the most likely hypothesis. Instead, because the experimental noise per experiment is estimated alongside the kinetic parameters, the zero-hypothesis yields unrealistically precise estimates, due to the algorithm indicating that under the assumption that this hypothesis is true, the experimental uncertainties are much larger. The four hypotheses that do model the influence product inhibition have similar precisions in their parameter estimates.

To compare all hypotheses and determine the most (and least) likely ones, we performed a Leave-one-out (LOO) cross-validation directly from the posterior probability distributions.^36^ This test used the Pareto Smoothed Importance Sampling (PSIS) approximation for most observed data points and exact LOO for data points where this approximation was not valid. This test efficiently determines the model that approximates the observed data best and possesses the highest predictive accuracy, while taking into consideration the complexity of the models (e.g., the number of kinetic parameters involved) to prevent overfitting. From this hypothesis comparison, we can conclude that given the experiments performed up to this point, a cooperative noncompetitive inhibition (*H*_4_) is the most likely product-inhibition mechanism occurring in the G6PDH PEBs, although the noncooperative variant (*H*_3_) cannot be ruled out. The zero-hypothesis cannot be ruled out completely, only requiring high experimental uncertainties to be explained by the data, although it remains unlikely. Both hypotheses for competitive inhibition can be ruled out with some confidence. Similarly, while the zero-hypothesis is likely not correct, a small chance exists that indeed one experiment does contain large experimental errors and is therefore unreliable.

Hypotheses comparisons can be a useful exploratory or diagnostic tool to evaluate which proposed reaction mechanisms are relevant and worth investigating further. However, numerous sources of error and uncertainty still exist in (PSIS-)LOO and similar cross-validation algorithms, making them susceptible to decreased robustness when comparing many models, or when little data are available.^36^ This should be taken into consideration when evaluating these results and considering new experiments to perform.

## Conclusion

We have demonstrated how a Bayesian approach toward analyzing enzymatic reaction networks allows for more accurate inference of the kinetics in these networks, while simultaneously taking into account any experimental or model-related uncertainties. Using this approach, we have shown how experimental data can be combined in one coherent framework, in order for us to correlate the findings in these experiments and improve the estimation of parameters, as well as outlier detection. This approach essentially allows us to continuously improve these estimates further by iteratively adding more experimental data to our models. Moreover, this means that any new experiment might have the potential to unlock more information from older experiments in the process, enabling much more efficient data gathering. Lastly, we have shown how this approach can be used to compare the likelihood of different reaction mechanism hypotheses. Comparing reaction mechanisms from a probabilistic perspective is a potentially powerful tool that can be used to make informed decisions about the next best experiments to perform when many different mechanisms are under consideration. Importantly, it can equally well be used in reanalyzing old data sets in light of newly discovered or proposed mechanisms, or when new data become available. However, care should be taken in interpreting the results from these comparisons as final conclusions. It is not a suitable method to make statements about the absolute truth of a hypothesis, as the test only checks the predictive power of each hypothesis relative to all other hypotheses under consideration. Therefore, if no correct reaction mechanism is included in the hypotheses, then it will also not be considered in the test.

Bayesian methods open up multiple new areas of possibilities for the design of more complex enzymatic reaction networks, and for systems chemistry in general. In addition to the findings presented here, significant potential exists in the usage of knowledge from literature for more informative and realistic prior distributions, such as informed log-normal or gamma distributions, which could improve the obtained estimates further, and could allow for direct comparison between new results and previous studies, as well as help in deciding initial experimental designs. Furthermore, more advanced hierarchical models and the inclusion of latent variables could potentially aid in discovering previously unknown interactions or hidden factors affecting the behavior of ERNs,^37^ from both a chemical point-of-view (allosteric effects, influence of pH) and an experimental point-of-view (systematic measurement errors, equipment deterioration). In principle, the methods discussed here could be combined with other network analysis techniques (both analytical and experimental) for enhanced network discovery or, additionally, used in combination with other machine learning techniques to enhance predictive capabilities. Finally, calculation of the full posterior probability distributions opens the door for determining optimal experimental designs.^38,39^ These designs could be aimed at a variety of different goals, such as experimental conditions for the maximum information gain for a certain kinetic parameter, but also the maximum production of a specific substrate or set of substrates, taking automatically into account any uncertainties that still exist about the behavior of these systems.

We do note that the methods introduced here are still computationally relatively expensive, and some of the sampling techniques are not yet suitable for every type of data. We have also limited our present study to small enzymatic networks at steady state. For larger networks, determining the steady state via numerical optimization may be less viable, and explicit solving of the full ODE system may be necessary. While inference on time-dynamic data is in principle possible, we found that current implementations are computationally demanding, while they do not yet significantly improve parameter estimates. This, however, is an area of active research that we hope can be improved in the future to allow for the inclusion of time-varying data and more complex models. Additionally, while our approach can indicate the presence of bad data and experimental errors, it does not guarantee the absence of sources of error. Care should still be taken to avoid a false sense of security when precise parameter estimates are obtained.

In conclusion, we have shown that the Bayesian approach we demonstrate here is highly relevant for the construction of complex enzymatic networks, allowing researchers to increase the predictability and reproducibility of artificial enzymatic networks, and allowing the field of enzymatic reaction networks to mature beyond toy models and proof-of-concepts.

## Supporting Information Available

Methods, overview of experiments and computational methods, and computational model details and sampling diagnostics (PDF)
