# Supplementary material for: A Bayesian Approach to Extracting Kinetic Information from Artificial Enzymatic Networks
Source: Anal Chem. 2022 May 12;94(20):7311–8. doi: 10.1021/acs.analchem.2c00659 (PMC9134183; doi:10.1021/acs.analchem.2c00659)
Supplement: Supplementary file 1 — ac2c00659_si_001.pdf [file ac2c00659_si_001.pdf]

Supporting Information - A Bayesian approach to extracting kinetic information from artificial enzymatic networks

Mathieu G. Baltussen[1] Jeroen van de Wiel[1] Cristina Lía Fernández Regueiro[1]
Miglė Jakštaitė[1] Wilhelm T.S. Huck[1,*]

[1] Institute for Molecules and Materials, Radboud University Nijmegen, 6525 AJ, Nijmegen, the Netherlands;

[*] Correspondence: Wilhelm T.S. Huck <w.huck@science.ru.nl>

Contents

Materials and Instrumentation **S3**
Materials . . . S3
Enzymes . . . S3
Substrates . . . S3
Instrumentation & Quantification protocols . . . S3
Flow setup . . . S3
Spectroscopy . . . S5
High-performance liquid chromatography . . . S5
G6P-DH assay . . . S5

Production and characterisation of polyacrylamide-enzyme beads **S6**
Microfluidic devices fabrication for beads production . . . S6
Method 1: Enzyme-first functionalization . . . S6
Synthesis 6-acrylaminohexanoic acid succinate (AAH-Suc) linker . . . S6
Enzyme functionalization . . . S6
General Enzyme Immobilization During Polymerization (EIDP) method . . . S6
Method 2: . . . S7
Empty bead production method . . . S7
General Enzyme Immobilisation after Polymerisation (EIAP) Method . . . S7
Overview of PEBs . . . S7

Overview of experiments **S9**

Overview of computational methods **S11**

Extended iterative combination of experiments **S11**

Model details & sampling diagnostics **S13**
Obtaining improved accuracy from correlated parameter estimates . . . S13
Model . . . S13
Diagnostics . . . S13
Combining diverse experimental datasets . . . S16
Model . . . S16
Diagnostics . . . S16

Comparing reaction mechanism hypotheses . . . S18
Models . . . S18
Hypothesis comparison . . . S18
Hypothesis 0 . . . S18
Hypothesis 1 . . . S19
Hypothesis 2 . . . S19
Hypothesis 3 . . . S19
Hypothesis 4 . . . S22

References **S28**

Materials and Instrumentation

Materials

All chemicals and reagents were used as received from commercial suppliers without any further treatment unless stated otherwise.

Enzymes

The enzymes immobilized on PEBs were purchased from Merck Sigma-Aldrich. Specifically:

- Lyophilized Trypsin (Tr) from bovine pancreas (product no. T1426)
- Lyophilized Glucose Dehydrogenase (GDH) from Pseudomonas sp. (product no. 19359)
- Lyophilized Hexokinase, type F-300 (HK) from Saccharomyces cerevisiae (product no. H4502)
- Lyophilized Glucose-6-phosphate dehydrogenase (G6P-DH) from Leuconostoc menesteroides (product no. G8529)

Trypsin reactions was conducted in a 200 mM Tris buffer, pH 7.8, with 20 mM of CaCl2. GDH, HK and G6P-DH reactions were conducted in a 200 mM Tris buffer, pH 7.8, with 10 mM of MgCl2.

Substrates

The substrates Cbz-Arg-AMC (R-AMC, CAS: 3701-04-6, New catnr: 4002540.0050) and Suc-Ala-Ala-Ala-AMC (AAA-AMC, CAS 73617-90-0, New catnr: 4006305.0050) were purchased from BioConnect B.V. and were dissolved as 150 mM stock in anhydrous DMSO and kept frozen at -20°C.

The substrates employed for all metabolic enzymatic reactions were:

- Glucose (anhydrous) (Merck product no. 1083370250)
- glucose-6-phosphate hydrate (Sigma Aldrich product no. G7250)
- ATP disodium salt (Sigma Aldrich product no. A26209)
- ADP monosodium salt (Sigma Aldrich product no. A2754)
- NAD+ (Roche product no. 10127965001)
- NADH disodium salt (Roche product no. 10107735001)
- NADP+disodium salt (Roche product no. 10128031001)
- NADPH tetrasodium salt (Roche product no. 1010724001)

Instrumentation & Quantification protocols

Flow setup

A custom made CSTR (see Figure S1 for the design schematic, effective volume $100\mu L$) was charged with PEBs (in a ratio of 1:31 mg beads:injection volume). The openings of the reactor were sealed with Whatman Nuclepore Track-Etch polycarbonate membranes (5 μm poresize, cat. number 10417414) to prevent outflow of PEBs. To subject the CSTR to various flow conditions we used Cetoni Low-Pressure High-Precision Syringe Pumps neMESYS 290N and gastight Hamilton syringes, with all flowrates programmed using the Cetoni neMESYS software. For all methods of offline detection, the outflow of the CSTR was connected to a BioRad 2210 Fraction collector, collecting for 7.5 (absorbance) or 10 minutes (HPLC) per fraction. This experimental setup is shown in Figure S2a-b

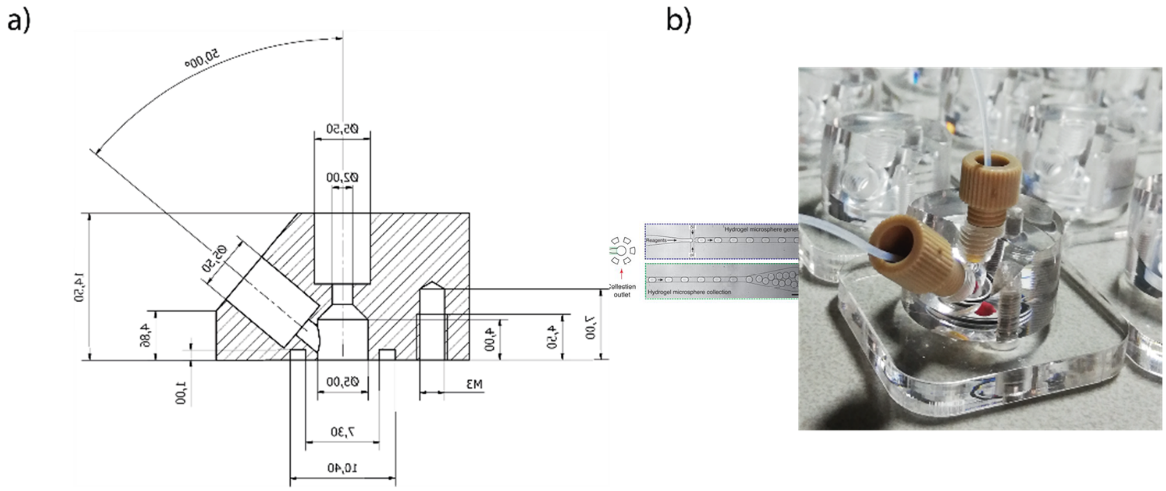

Figure S1: (a) Schematic overview of the CSTR. (b) Photograph of the CSTR

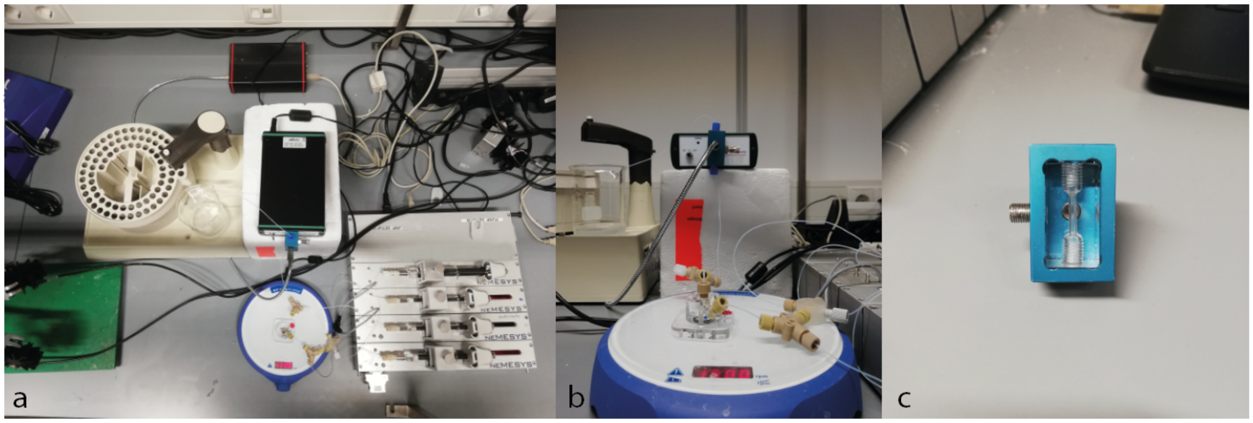

Figure S2: (a) Overview of the experimental setup, with left the fraction collection, lower center the CSTR on a stirring plate, and lower right the neMESYS pump system. (b) Close-up of the CSTR connected to input and output tubing. (c) Close-up of the flow cuvette used for online absorbance detection, provided by LabM8

Spectroscopy

Offline fluorescence Collected fractions were pipetted onto a microplate (Greiner Bio One, black, polystyrene, 96 flat bottom chimney wells) at 60 μL per well. Fluorescence measurements for enzyme activity determination were performed with a Tecan Spark 10M plate reader. The fluorescence intensity of wells containing 30-200 μL of the reaction mixture was monitored for 1-4 min (shaking 3s/orbital mode/amplitude 4mm) at 23 °C using top or bottom reading mode, at $\lambda_{ex}/\lambda_{e}m$ = 380nm/460nm for 7-amino-4-methylcoumarin (AMC) based substrates.

Offline absorbance Collected fractions were pipetted onto a microplate (Greiner Bio One, transparent, polystyrene, 96 flat bottom chimney wells) at 60 μL per well. The absorbance of each sample at 340 nm was measured in a TECAN SPARK M10 platereader. Using a calibration curve, the absorbance data was converted to NADH concentration.

Online absorbance Absorbance in flow experiments was continuously measured at the reactor's output with a custom made flow cell kindly provided to us by LabM8 (shown in Figure S2c), connected to an AvaLight 355 nm LED lamp. Absorbance at 380 nm was detected using an AvaSpec-2048 with 1,005 ms integration time and averaging at 200 times per recorded datapoint. Using a calibration curve, the absorbance data was converted to NADH concentration.

High-performance liquid chromatography

High-performance Liquid Chromatography (HPLC) was performed using Shimadzu NexeraX3/Prominence system under a 0.9 mL/min flow at 45 °C with a Shimadzu WAX-1 column. For the Shimadzu system, an injection volume was subjected to a 25 min gradient program was used starting from 20 mM of potassium phosphate at pH 7.0

- 1 min – 20 mM at pH 7.0
- 16 min – 480 mM at pH 6.8
- 19 min – 480 mM at pH 6.8
- 22 min – 20 mM at pH 7.0
- 25 min – 20mM at pH 7.0

Retention time of ADP is at 8.8 minutes, ATP at 15.5 minutes.

G6P-DH assay

Collected fractions were pipetted onto a microplate (Greiner Bio One, transparent, polystyrene, 96 flat bottom chimney wells) at 60 μL per well. To these wells was added an 20 μL of 5 mM of NADP+ and 1 μL of 500 u/mL of G6P-DH. The absorbance of each sample at 340 nm was continuously measured in a TECAN SPARK M10 platereader. Using a calibration curve, the absorbance data was converted to NADPH concentration. We assumed this NADPH concentration equivalent to G6P concentration.

Production and characterisation of polyacrylamide-enzyme beads

Microfluidic devices fabrication for beads production

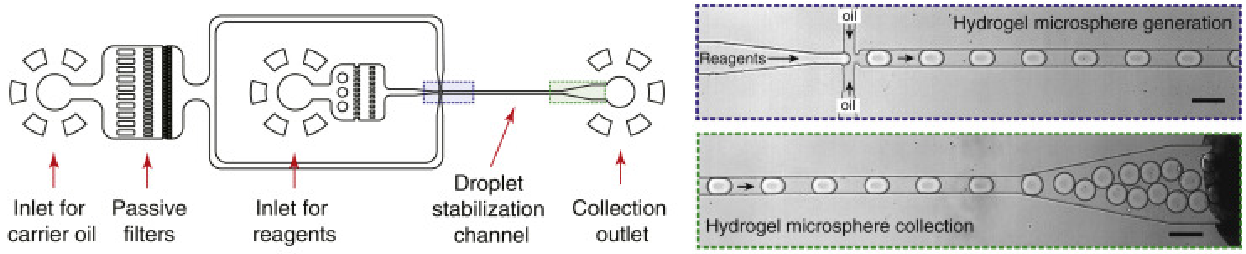

Figure S3: Schematic of the microfluidic device for droplets production (*adaptated from*[1])

Microfluidic devices, designed according to the scheme shown in Figure S3[1], were produced in silicon wafers by photo and soft lithography, with different orifice sizes (20-80μm) at the T-junction, for droplet formation. After that, the wafers were used for the production of polydimethylsiloxane (PDMS) devices. The PDMS replicas were separated from the wafer, and inlets and outlets of 1mm inner diameter were punched. After the replica was bonded by oxygen plasma treatment to a glass slide, channels were coated with 2% 1H,1H,2H,2H-Perfluoro-octlytriethoxysilane to make them hydrophobic[2].

Method 1: Enzyme-first functionalization

This method was used for the synthesis of PEBs containing Trypsin.

Synthesis 6-acrylaminohexanoic acid succinate (AAH-Suc) linker

N-6-acryloyl amido hexanoic acid (30 mg, 0.172 mmol, 1 eq) and N-Hydroxy succinimide (20mg, 0.172 mmol, 1eq) were added in a large glass vial and brought under N2 atmosphere. Subsequently, they were dissolved in 1 mL of anhydrous DMF and cooled down to 0°C. Then, N, N'-dicyclohexylcarbodiimide (DCC, 38mg, 0.172mmol, 1eq) was added, and the vial was sealed and left to stir at 0°C for one hour. Subsequently, the reaction was brought to 4°C and stirred for 16h. The reaction mixture was assumed quantitative and immediately used for enzyme functionalisation without further analysis[3].

Enzyme functionalization

The desired enzyme (1 eq) was added to a falcon tube containing 0.1 M NaHCO3 (4mL), followed by addition of AAH-Suc (7.5 eq) in DMF. The mixture was left to stir at 21 °C for 1 h, after which the reaction mixture was dialysed and lyophilised.

General Enzyme Immobilization During Polymerization (EIDP) method

An emulsion of monodisperse water-in-oil droplets was produced by using the microfluidic device described above with a 20 μm-wide T-junction, following an adapted procedure from Rivello et al.[3] The reagents phase used for hydrogel formation was composed by 9.7% (w/v) acrylamide, 0.4% (w/v) bisacrylamide, 1.5% (w/v) 2,2'Azobis(2-methylpropionamidine) dihydrochloride (AAPH) and 0-8% (v/v) of a solution of functionalized enzyme (100 μM). The oil phase was composed of fluorinated fluid HFE-7500 (3M) and 1.5% (v/v) Pico-Surf™ 1. The flow rates used to produce monodisperse beads of 50μm average diameter were 600$\mu L/h$ (Qw) for the reagents phase and 900$\mu L/h$ for the oil phase (Qo). The emulsion of droplets created

in the microfluidic device was collected in an Eppendorf with 100 uL mineral oil to avoid evaporation and breaking the emulsion and polymerised for 10 minutes under UV light. After polymerisation, beads were washed three times with 20% (v/v) 1H,1H,2H,2H perfluorooctanoic (PFO) in HFE-7500 oil to break the emulsion. The obtained beads were then washed three times with 1% (v/v) Span 80 in heptane, three times with 0.1% (v/v) Triton X-100 in miliQ and three times with Milli-Q. After every washing step, the beads were centrifugated 30 seconds at 5000 rcf, and the supernatant was removed by pipetting. The resulting beads were freeze-dried and re-dissolved in miliQ at a concentration of 0.0322mg/μL. Bead size was obtained after freeze-drying by imaging with a light microscope with a 40x objective lens. The average bead size was 50 μm.

Method 2:

This method was used for the synthesis of PEBs containing GDH, HK, and G6PDH.

Empty bead production method

The microfluidics device described above was used to produce gel beads with a 20 μm wide T-junction, following an adapted procedure from[2]. The gel solution phase consisted of 9.6% (w/v) acrylamide, 0.4% (w/v) N,N'-methylenebisacrylamide, 0.5% (w/v) acrylic acid and 1.5% (w/v) 2,2'Azobis(2-methylpropionamidine) dihydrochloride. The oil phase contained 1.5% (v/v) Pico-Surf™ 1 in fluorinated fluid HFE-7500 (3M). The flow rates for gel phase and oil phase were 600 $\mu L/h$ and 900 $\mu L/h$, respectively. The outflow emulsion was collected in the tube which was filled with 100 μL mineral oil. Afterwards beads were polymerised using UV lamp for 10 minutes at 70% gain. After polymerisation the lowest layer containing fluorocarbon phase was carefully removed with a P200 pipette. The remaining beads were washed 3 times with 20% (v/v) 1H,1H,2H,2H-Perfluoro-1-octanol in HFE-7500 (3M), then 3 times with 1% (v/v) Span 80 in hexane, 3 times with 0.1% (v/v) Triton X-100 in Milli-Q and finally 3 times with Milli-Q. Every washing step was finalised with mixing the tube using vortex, centrifuging at 5000 x g for 3 min and removing the layer which was not containing beads. Furthermore, beads were flash frozen using nitrogen and freeze dried overnight. After re-wetting beads, their size was determined using light microscope with 40x magnitude objective. The average size was 50 μm in diameter.

General Enzyme Immobilisation after Polymerisation (EIAP) Method

Empty acrylamide beads were re-dissolved in Milli-Q at a concentration of 0.0322 mg/μL. 1-(3-Dimethylaminopropyl)-3-ethylcarbodiimide hydrochloride (100 mM) and N-Hydroxysuccinimide (100 mM) were added to the reaction mixture. The total volume of the activation solution was 5-fold of the beads volume. The reaction mixture tube was put on the roller bank for 30 min. After this, beads were centrifuged at 5000 x g for 3 min. The supernatant was carefully removed using P200 pipette. Beads were washed 3 times by adding Milli-Q, mixing using vortex, centrifuging and removing the supernatant. Specific to each batch of PEBs, a certain amount of enzyme was added to the beads (see Table S1). The tube was put on the roller bank for 2 h coupling step. Sequentially, beads were washed 8 times by adding Milli-Q, centrifuging and removing the supernatant. Finally, beads were flash frozen using nitrogen and freeze dried overnight.

Overview of PEBs

Table S1: Overview of PEB batches

Enzyme	Batch number	Enzyme concentration added	Volume per mg of beads
GDH	1	0.54 kUnit/mL	100 μL/mg
	2	2 kUnit/mL	100 μL/mg

Enzyme	Batch number	Enzyme concentration added	Volume per mg of beads
	3	1 kUnit/mL	100 μL/mg
HK	1	1 kUnit/mL	100 μL/mg
G6P-DH	1	1 kUnit/mL	100 μL/mg

Overview of experiments

Processed datafiles from these experiments can be found on the accompanying github repository in the `data` folder as csv-files. The files are directly used during analysis performed in the Jupyter notebooks.

In the tables below, the volume refers to the volume of PEBs suspension injected into the CSTR, where the PEBs suspension itself is created by suspending 1 mg of dry PEBs in 31 μL buffer, or other quantities in the same ratio (1:31). The batch refers to the batch of PEBs, which can contain different effective enzyme concentation. See Table S1 for an overview of all PEB batches.

Table S2: Overview of single-enzyme experiments

Code	flowrate ($\mu L/h$)	enzyme	volume (μL)	batch	inputs	observables	observation techniques
SNCA14	750	GDH	10	1	G, NAD	NADH	offline abs.
SNCA15	750	GDH	10	1	G, NAD	NADH	offline abs.
SNCA17	750	HK	10	1	G, ATP	G6P	G6P assay
SNCA18	750	HK	1	1	G, ATP	G6P	G6P assay
SNKS03	750	HK	1	1	G, ATP	G6P	G6P assay
SNKS04	750	HK	1	1	G, ATP	G6P,ATP	G6P assay, HPLC
SNKS08	750	G6PDH	10	1	G6P, NAD	NADH	offline abs.
SNKS11	750	GDH	0.5	2	G, NAD	NADH	online abs.
SNKS12	750	GDH	0.5	2	G, NAD	NADH	online abs.
SNKS18	750	GDH	2.0	3	G, NAD	NADH	offline abs.
SNKS20	750	G6PDH	2.0	1	G6P, NAD	NADH	online abs.

Table S3: Overview of multi-enzyme experiments

Code	flowrate ($\mu L/h$)	enzymes	volumes (μL)	batches	inputs	observables	observation techniques
SNKS06	750	GDH,HK	10.0, 1.0	1, 1	G, NAD, ATP	NADH, ADP	online abs., HPLC
SNNS002	750	GDH,HK	0.5, 1.0	2, 1	G, NAD, ATP	NADH	online abs.
SNNS003	750	GDH,HK	0.25, 1.5	2, 1	G, NAD, ATP	NADH	online abs.
SNNS004	750	GDH,HK	0.333, 1.33	2, 1	G, NAD, ATP	NADH	online abs.
SNNS005	750	GDH,HK	0.666, 0.666	2, 1	G, NAD, ATP	NADH	online abs.
SNNS006	750	GDH,HK	0.75, 0.5	2, 1	G, NAD, ATP	NADH	online abs.
SNNS007	750	GDH,HK	0.5, 1.0	2, 1	G, NAD, ATP	NADH	online abs.

Overview of computational methods

Python scripts and Jupyter notebooks were used to create the Bayesian models and perform inference and predictive sampling. All computational studies were performed with Jupyter notebooks. Datasets were loaded in from csv-files with Pandas, and if relevant, concatenated together into larger objects.

The Bayesian model, including the determination of prior probabilities and likelihood function, was created using PyMC3[4]. Generally, prior probabilities for Michaelis-Menten parameters were chosen as uniform distributions over a specified interval. These distributions were used as uninformative priors to ensure no subjective information would enter the model, while garanteeing correct sampling and estimation of parameters. Priors for the uncertainty estimations (denoted by `sigma` in the notebooks) were given an exponential distribution, which also acted as an uninformative distribution, while garanteeing correct sampling and convergence. In larger models, where multiple likelihoods were combined, hyperpriors were placed on the k_{cat} and σ priors to increase convergence of the sampling algorithm.

All sampling was peformed using the No-U-Turn Sampler (NUTS)[5], which is an adaptive step-size Hamiltonian Monte Carlo sampler. When (automatically, or via a custom operator) the gradients of the likelihoods with respect to the kinetic parameters were given, this sampling method is much more efficient then a classical Metropolis Monte Carlo sampler, showing faster convergence and requiring less samples for precise posterior estimations. Generally, sampling was performed using 4 or 8 independent chains on 4 or 8 cpu cores, all with 1000 tuning steps, and 1000 sampling steps, and a target step acceptance probability of 0.95. These values were found to yield good sampling results without becoming computatially inefficient.

For likelihood calculations of partially observable ERNs, a custom steady-state likelihood operator was written in Theano, according to description given in the manuscript. This operator is freely available from the public github repository at https://github.com/mgbaltussen/BayERN.

The samples obtained from the posterior distribution were further analysed using standard statistical tools in Python, the Numeric Python package and the Scientific Python package (NumPy and Scipy)[6]. To ensure the accessibility and reproducibility of these results the datasets and Jupyter notebooks, used for the analysis and creation of figures found in the publication, are made available as additional Supporting Information, and directly on github at https://github.com/huckgroup/Bayesian-enzymatic-networks_manuscript_2022. This repository also includes three example notebooks with additional explanation (`example_simulation.ipynb`, `example_simple.ipynb`, and `example_complex.ipynb`) and version information for all software dependencies.

Extended iterative combination of experiments

See Figure S4 for an extended version of the figure, showing posterior updating per iteration of new experiment.

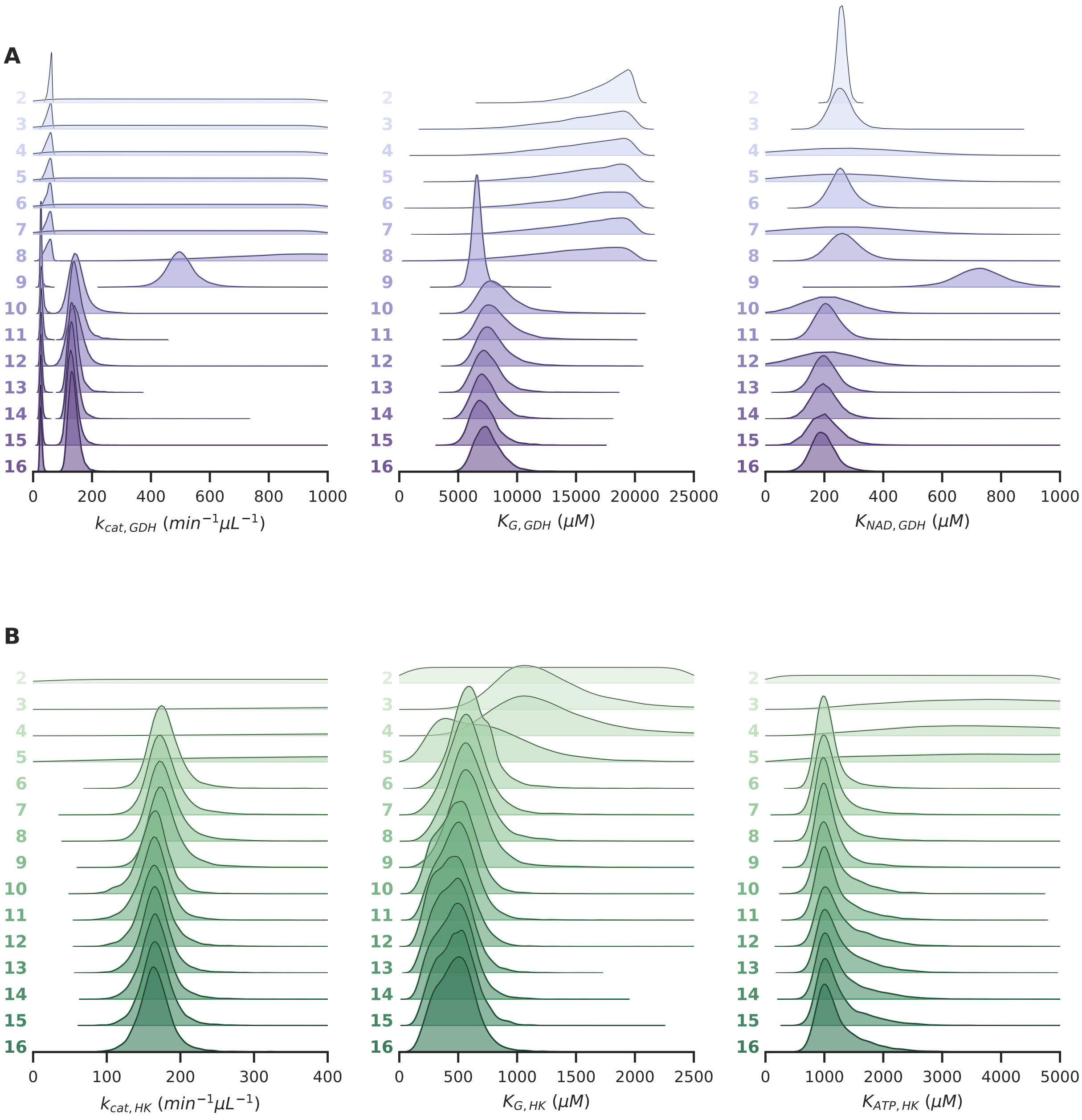

Figure S4: **Extended iterative posterior updating** (a,b) Posterior parameter estimates obtained from the model combining all three (GDH, HK, GDH+HK) observation likelihoods. For every parameter, the distributions are shown for 15 different datasets, with each following dataset containing an extra experiment, added in chronological order. Distributions are shifted and scaled to increase visibility. For the GDH k_{cat}, two estimates are obtained because PEBs with two different enzyme concentrations were used in different experiments.

Model details & sampling diagnostics

In the following section, the mathematical models on which the analysis is based are described in more detail, alongside additional information on sampling statistics and diagnostics. All three sections have a corresponding Jupyter notebook on the github repository at https://github.com/huckgroup/Bayesian-enzymatic-networks_manuscript_2022.

Obtaining improved accuracy from correlated parameter estimates

Model

The cleavage of R-AMC to AMC by the peptidase Trypsin was modelled following a Michaelis-Menten rate equation with an additional term describing uncompetitive inhibition by the inhibitor AAA-AMC. This results in the following system of ODE's:

$$\mathbf{f}(\mathbf{C}, \phi, \theta) : \begin{cases} \frac{d[P]}{dt} &= \frac{E \cdot k_{cat} \cdot [S]}{K_M + [S](1+[I]/K_I)} - k_f[P] \\ \frac{d[S]}{dt} &= \frac{-E \cdot k_{cat} \cdot [S]}{K_M + [S](1+[I]/K_I)} + k_f([S]_{in} - [S]) \end{cases}$$

where $[S]$ is the concentration of the substrate R-AMC, $[P]$ the concentration of the product AMC, and $[I]$ the concentration of the inhibitor AAA-AMC. This system contains 3 kinetic parameters that need to be inferred, $\phi = \{k_{cat}, K_M, K_I\}$, and 4 control parameters $\theta = \{E, k_f, [S]_{in}, [I]\}$. The measurements of the observed product concentration are assumed to have normal-distributed noise $[P]_{obs} \sim N([P]_{ss}, \sigma)$ with a mean equal to the true steady-state concentration and an unknown standard-deviation σ. As priors for all 3 kinetic parameters, uninformative uniform distributions are used, with the following upper and lower boundaries:

$$P(k_{cat}) = \mathcal{U}(0, 500)$$
$$P(K_M) = \mathcal{U}(0, 500)$$
$$P(K_I) = \mathcal{U}(1000, 10000)$$

As prior for the noise estimate sigma, an uninformative exponential distribution is used:

$$P(\sigma) = \text{Exp}(10)$$

The full posterior is described by

$$\begin{aligned} P\left(k_{cat}, K_M, K_I, \sigma \middle| [P]_{obs}, E, k_f, [S]_{in}, [I]\right) &\propto \prod_i P(\phi_i)\mathcal{L}(y, \phi) \\ &\propto P(k_{cat})P(K_M)P(K_I)P(\sigma)\mathcal{N}\left([P]_{ss} - [P]_{obs}, \sigma\right) \end{aligned}$$

where $[P]_{ss} = g(\phi, \theta)$ is the steady-state solution of $\mathbf{f}(\mathbf{C}, \phi, \theta)$.

Diagnostics

Table S4: Sampling statistics for manuscript Figure 1

	Chains	Tuning steps	Draws	Mean acceptance rate	Divergences
0	4	1000	1000	0.948	0

Table S5: Sampling diagnostics for manuscript Figure 1

	mcse_mean	mcse_sd	ess_bulk	ess_tail	r_hat
k_cat	0.09	0.06	1206	1598	1
K_M	0.28	0.2	1236	1796	1
K_I	15.84	11.47	1557	1269	1
sigma	0	0	1657	2034	1

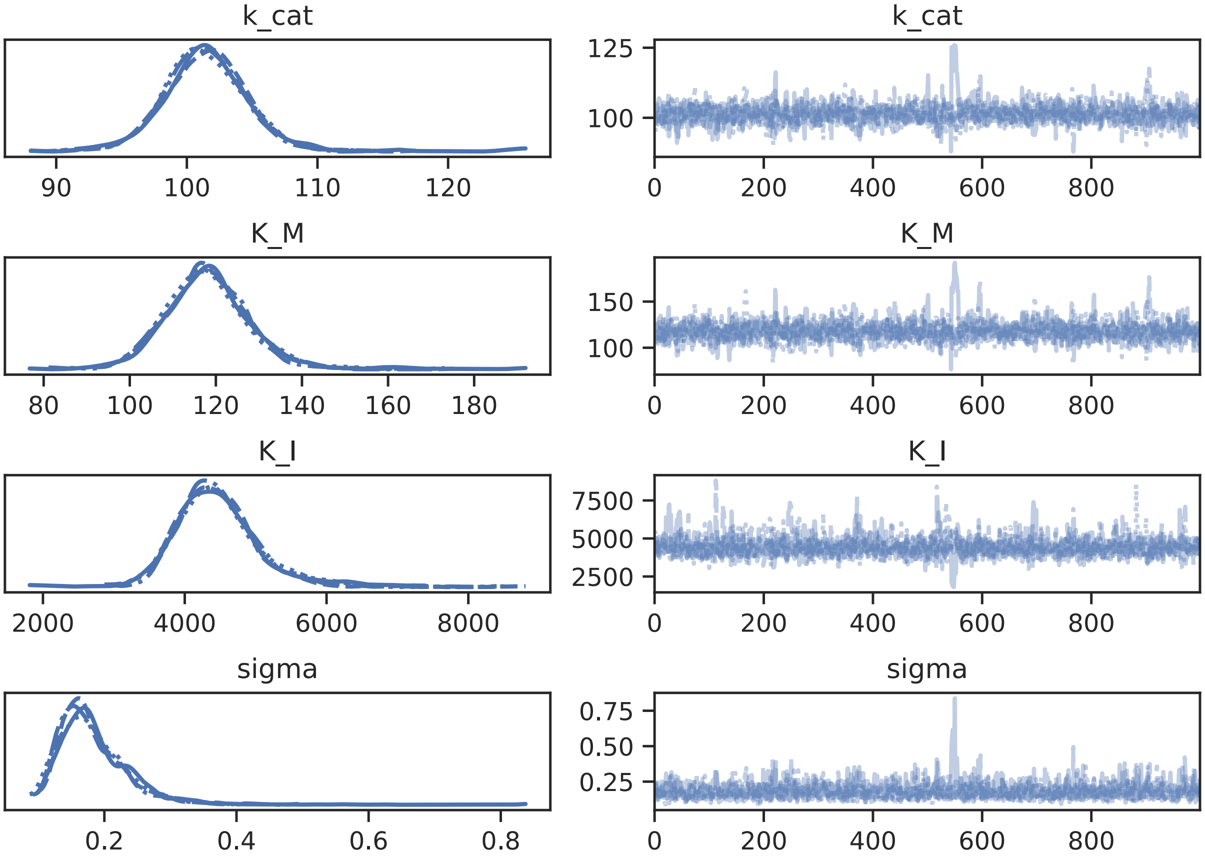

Figure S5: Diagnostic sampling trace plots of all kinetic parameters inferred in manuscript Figure 1

Combining diverse experimental datasets

Model

The system of GDH and HK PEBs was modeled as a competitive two-reaction system, where GDH catalyses the reaction G+NAD → GdL+NADH, and HK catalyses G+ATP → G6P+ADP. This system is described by the following set of ODE's:

$$\mathbf{f}(\mathbf{C}, \phi, \theta) : \begin{cases} \frac{d[G]}{dt} & = -\frac{[GDH] \cdot k_{cat}^{GDH} \cdot [G][NAD]}{1+[G]/K_G^{GDH}+[NAD]/K_{NAD}^{GDH}} - \frac{[HK] \cdot k_{cat}^{HK} \cdot [G][ATP]}{1+[G]/K_G^{HK}+[ATP]/K_{ATP}^{HK}} - k_f([G] - [G]_i n) \\ \frac{d[GdL]}{dt} & = \frac{[GDH] \cdot k_{cat}^{GDH} \cdot [G][NAD]}{1+[G]/K_G^{GDH}+[NAD]/K_{NAD}^{GDH}} - k_f[P] - k_f[GdL] \\ \frac{d[G6P]}{dt} & = \frac{[HK] \cdot k_{cat}^{HK} \cdot [G][ATP]}{1+[G]/K_G^{HK}+[ATP]/K_{ATP}^{HK}} - k_f[G6P] \\ \frac{d[NAD]}{dt} & = -\frac{[GDH] \cdot k_{cat}^{GDH} \cdot [G][NAD]}{1+[G]/K_G^{GDH}+[NAD]/K_{NAD}^{GDH}} - k_f([NAD] - [NAD]_{in}) \\ \frac{d[NADH]}{dt} & = \frac{[GDH] \cdot k_{cat}^{GDH} \cdot [G][NAD]}{1+[G]/K_G^{GDH}+[NAD]/K_{NAD}^{GDH}} - k_f[NADH] \\ \frac{d[ATP]}{dt} & = -\frac{[HK] \cdot k_{cat}^{HK} \cdot [G][ATP]}{1+[G]/K_G^{HK}+[ATP]/K_{ATP}^{HK}} - k_f([ATP] - [ATP]_{in}) \\ \frac{d[ADP]}{dt} & = \frac{[HK] \cdot k_{cat}^{HK} \cdot [G][ATP]}{1+[G]/K_G^{HK}+[ATP]/K_{ATP}^{HK}} - k_f[ADP] \end{cases}$$

Depending on the experiment, either the $[NADH]$ concentration or the $[ATP]$ concentration at steady-state are observed, from which the 6 kinetic parameters are inferred, $\phi = \{k_{cat}^{GDH}, K_G^{GDH}, K_{NAD}^{GDH}, k_{cat}^{HK}, K_G^{HK}, K_{ATP}^{HK}\}$. In every experiment, 6 control parameters exist: $\theta = \{[GDH], [HK], k_f, [G]_{in}, [NAD]_{in}, [ATP]_{in}, \}$. The measurements of the observed product concentration are assumed to have normal-distributed noise $[P]_{obs} \sim N([P]_{ss}, \sigma)$ with a mean equal to the true steady-state concentration and an unknown standard-deviation σ. As priors for the 4 Michaelis kinetic parameters, uninformative uniform distributions are used, with the following upper and lower boundaries:

$$\begin{aligned} P(K_G^{GDH}) &= \mathcal{U}(1, 20000) \\ P(K_{NAD}^{GDH}) &= \mathcal{U}(1, 20000) \\ P(K_G^{HK}) &= \mathcal{U}(1, 4000) \\ P(K_{ATP}^{HK}) &= \mathcal{U}(1, 6000) \end{aligned}$$

The 0-value was not included in the domain of these priors because it is highly unlikely and causes problems with divergent sampling.

The turn-over number k_{cat} is not only different for both enzymes, but can also vary between batch of enzymes or PEB's. To somewhat constrain the inferred values and ensure that the sampler can properly converge, the priors were modelled as uniform distributions with an upper boundary sampled from a gamma-distribution.

As priors for the noise estimate sigmas for each experiment, an exponential distribution was used. To help the sampling procedure, an exponential hyperprior was used for the distribution parameters.

Diagnostics

Table S6: Sampling statistics for manuscript Figure 3

	Chains	Tuning steps	Draws	Mean acceptance rate	Divergences
0	8	1000	1000	0.974	5

Table S7: Sampling diagnostics for manuscript Figure 3

	mcse_mean	mcse_sd	ess_bulk	ess_tail	r_hat
k_cat_hyper	10.3	7.29	1163	1714	1
k_GDH_cat[1]	0.07	0.05	4374	3463	1
k_GDH_cat[2]	0.31	0.23	4159	3295	1
k_GDH_cat[3]	0.06	0.04	4464	3944	1
k_HK_cat	0.47	0.39	4972	2798	1
K_GDH_G	20.52	15.48	4518	3262	1
K_GDH_NAD	0.75	0.62	4937	4050	1
K_HK_G	3.5	2.47	2120	3584	1
K_HK_ATP	13.33	11.48	2030	2290	1
sigma	0	0	7195	5833	1
sigma_HK[SNCA17]	1.39	0.98	2462	3719	1
sigma_HK[SNCA18]	1.44	1.08	7794	5116	1
sigma_HK[SNKS03]	0.58	0.41	2484	4945	1
sigma_HK[SNKS04]	0.77	0.55	2726	4230	1
sigma_GDH[SNCA14]	0.48	0.39	6051	3925	1
sigma_GDH[SNCA15]	0.3	0.22	6236	4164	1
sigma_GDH[SNKS11]	0.48	0.38	8520	4796	1
sigma_GDH[SNKS12]	0.5	0.39	8737	5330	1
sigma_GDH[SNKS18]	0.11	0.08	5624	4523	1
sigma_HK_GDH_1[SNKS06]	1.22	0.91	8526	5309	1
sigma_HK_GDH_2[SNNS002]	0.02	0.01	4710	4867	1
sigma_HK_GDH_2[SNNS003]	0.19	0.14	7393	3944	1
sigma_HK_GDH_2[SNNS004]	0.06	0.05	6504	4159	1
sigma_HK_GDH_2[SNNS005]	0.2	0.15	7370	4777	1
sigma_HK_GDH_2[SNNS006]	0.44	0.34	8358	3739	1
sigma_HK_GDH_2[SNNS007]	0.16	0.12	8927	5406	1

Comparing reaction mechanism hypotheses

Models

The system of G6PDH PEB's was modeled as the reaction G6p + NAD → G6PdL + NADH. Every hypothesis under investigation followed a different proposed rat equation, but the priors were kept as equivalent uniformative priors for every hypothesis:

$$P(k_{cat}) = \mathcal{U}(0, 500)$$
$$P(K_{NAD} = \mathcal{U}(1, 4000)$$
$$P(K_{G6P}) = \mathcal{U}(1, 2000)$$
$$P(K_{I,NADH}) = \mathcal{U}(1, 10000)$$

As prior for the noise estimate sigma, an uninformative exponential distribution was used:

$$P(\sigma) = \mathrm{Exp}(0.5)$$

Hypothesis comparison

Comparison of the predictive value of each hypothesis was done based on a PSIS-LOO cross-validiation calculation, with exact LOO cross-validation for observations where the Pareto shape exceeded 0.5. The exact LOO was performed by by direct resampling of the model with the selected observation left out. Results of this procedure can be found in the table below.

Table S8: Mechanism comparison diagnostics for PSIS-LOO cross-validation, with exact LOO performed for observations where the PSIS-LOO approximation is invalid.

	rank	loo	p_loo	d_loo	weight	se	dse	warning	loo_scale	n_eloo
H_4	0	-254.626	8.60706	0	0.551124	13.7688	0	False	log	3
H_3	1	-256.188	8.60793	1.56206	1.28014e-16	14.014	1.15425	False	log	5
H_1	2	-259.648	9.48013	5.02224	0	14.6769	3.11986	False	log	3
H_2	3	-264.265	9.8864	9.63904	6.49712e-15	13.8464	3.56142	False	log	2
H_0	4	-271.656	9.64548	17.0302	0.448876	23.1551	15.3058	True	log	3

Hypothesis 0

Table S9: Sampling statistics for manuscript Figure 5, Hypothesis 0

	Chains	Tuning steps	Draws	Mean acceptance rate	Divergences
0	4	1000	1000	0.929	0

Table S10: Sampling diagnostics for manuscript Figure 5, Hypothesis 0

	mcse_mean	mcse_sd	ess_bulk	ess_tail	r_hat
k_cat	0.06	0.04	1695	1703	1
K_G6P	1.58	1.12	1764	1914	1
K_NAD	0.61	0.43	2663	2020	1
sigma[SNKS08]	0.3	0.21	2763	2443	1

	mcse_mean	mcse_sd	ess_bulk	ess_tail	r_hat
sigma[SNKS20]	0.03	0.02	2353	1959	1

Hypothesis 1

Table S11: Sampling statistics for manuscript Figure 5, Hypothesis 1

	Chains	Tuning steps	Draws	Mean acceptance rate	Divergences
0	4	1000	1000	0.916	0

Table S12: Sampling diagnostics for manuscript Figure 5, Hypothesis 1

	mcse_mean	mcse_sd	ess_bulk	ess_tail	r_hat
k_cat	0.19	0.14	1596	1953	1
K_G6P	4.25	3.01	1620	1965	1
K_NAD	0.93	0.66	2172	2181	1
KI_NADH	4.23	2.99	1606	2128	1
sigma[SNKS08]	0.28	0.2	2173	2218	1
sigma[SNKS20]	0.03	0.02	2395	1997	1

Hypothesis 2

Table S13: Sampling statistics for manuscript Figure 5, Hypothesis 2

	Chains	Tuning steps	Draws	Mean acceptance rate	Divergences
0	4	1000	1000	0.923	0

Table S14: Sampling diagnostics for manuscript Figure 5, Hypothesis 2

	mcse_mean	mcse_sd	ess_bulk	ess_tail	r_hat
k_cat	0.23	0.17	1194	1816	1
K_G6P	3.03	2.15	1818	1937	1
K_NAD	1.72	1.22	1304	1642	1
KI_NADH	7.39	5.28	1532	1661	1
sigma[SNKS08]	0.26	0.18	2417	2385	1
sigma[SNKS20]	0.04	0.03	1819	1766	1

Hypothesis 3

Table S15: Sampling statistics for manuscript Figure 5, Hypothesis 3

	Chains	Tuning steps	Draws	Mean acceptance rate	Divergences
0	4	1000	1000	0.924	0

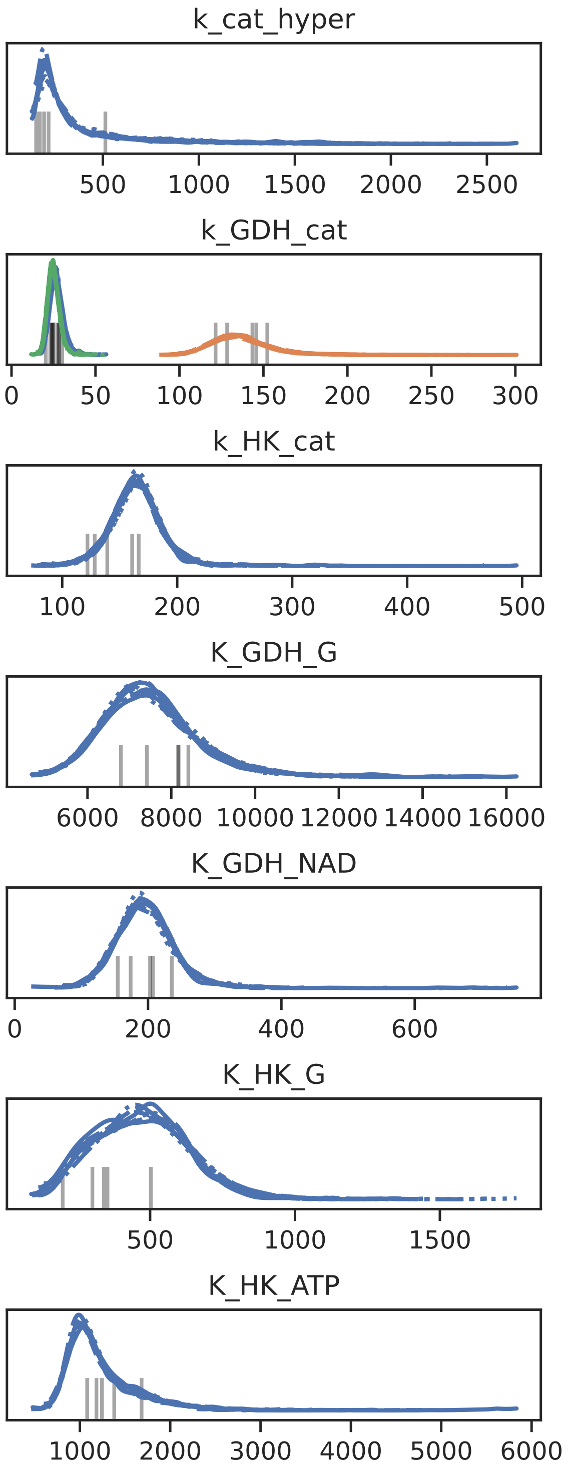

Figure S6: Diagnostic sampling trace plots of all kinetic parameters inferred in manuscript Figure 3

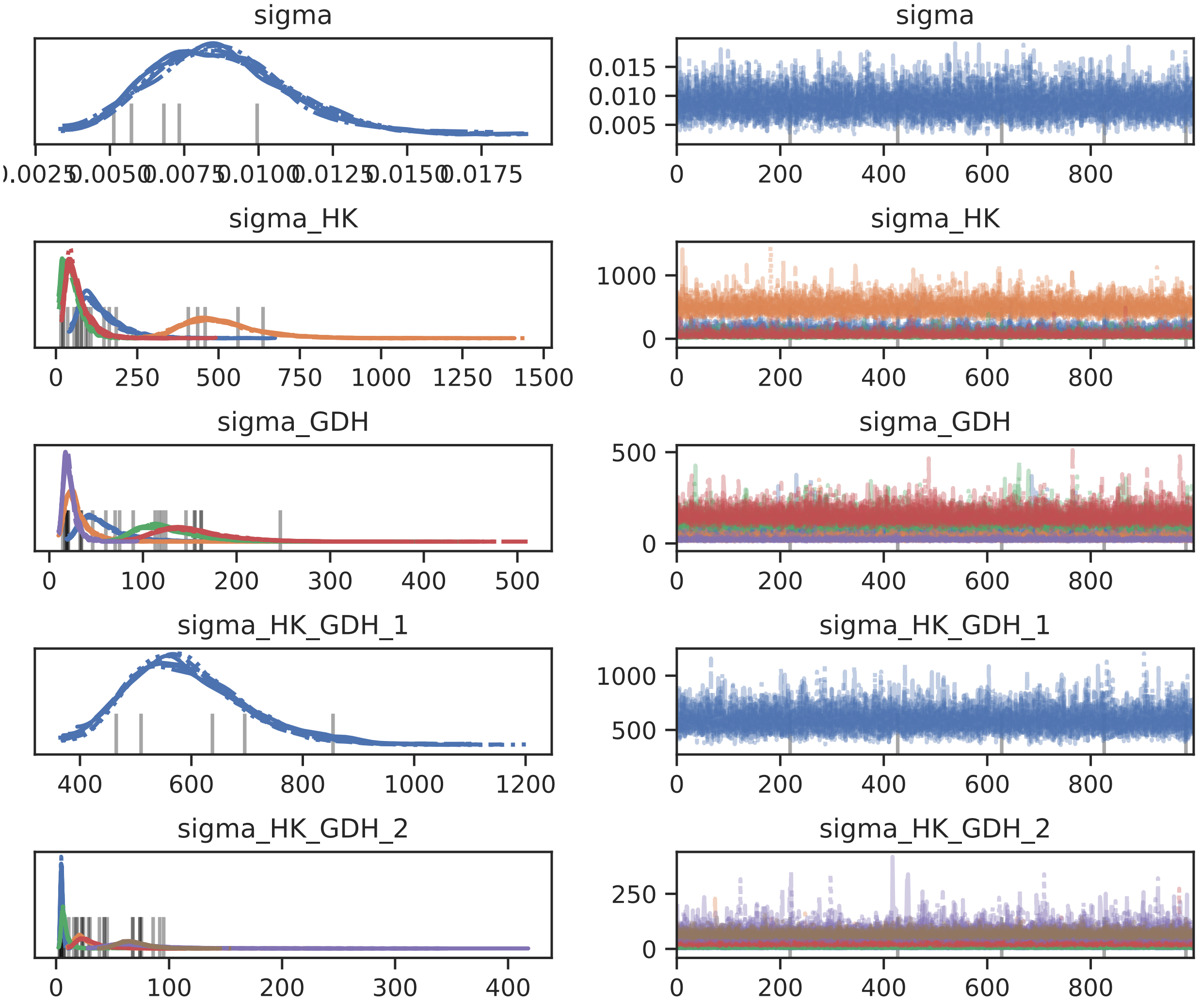

Figure S7: Diagnostic sampling trace plots of all uncertainty estimates inferred in manuscript Figure 3

Table S16: Sampling diagnostics for manuscript Figure 5, Hypothesis 3

	mcse_mean	mcse_sd	ess_bulk	ess_tail	r_hat
k_cat	0.39	0.28	939	1244	1
K_G6P	5.3	3.77	1093	1445	1
K_NAD	1.52	1.08	1377	1739	1
KI_NADH	10.55	7.46	1073	1791	1
sigma[SNKS08]	0.27	0.19	2270	2324	1
sigma[SNKS20]	0.04	0.03	1877	1748	1

Hypothesis 4

Table S17: Sampling statistics for manuscript Figure 5, Hypothesis 4

	Chains	Tuning steps	Draws	Mean acceptance rate	Divergences
0	4	1000	1000	0.924	0

Table S18: Sampling diagnostics for manuscript Figure 5, Hypothesis 4

	mcse_mean	mcse_sd	ess_bulk	ess_tail	r_hat
k_cat	0.33	0.24	1121	1285	1
K_G6P	4.7	3.35	1321	1226	1
K_NAD	1.41	1	1472	1679	1
KI_NADH	17.48	12.39	1258	1706	1
sigma[SNKS08]	0.24	0.18	2459	2625	1
sigma[SNKS20]	0.03	0.02	1867	2117	1

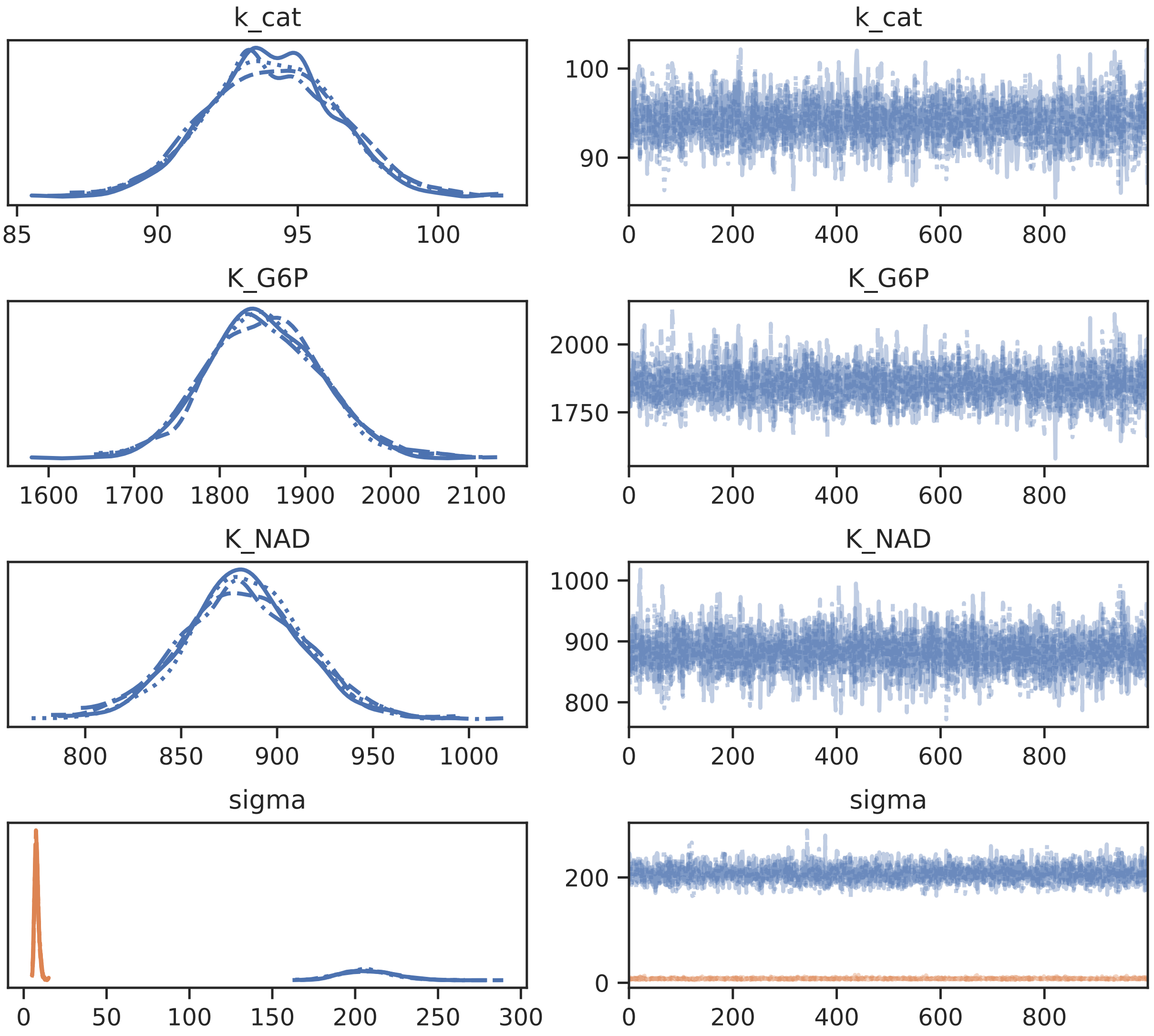

Figure S8: Diagnostic sampling trace plots of kinetic parameters inferred in manuscript Fig. 5, Hypothesis 0

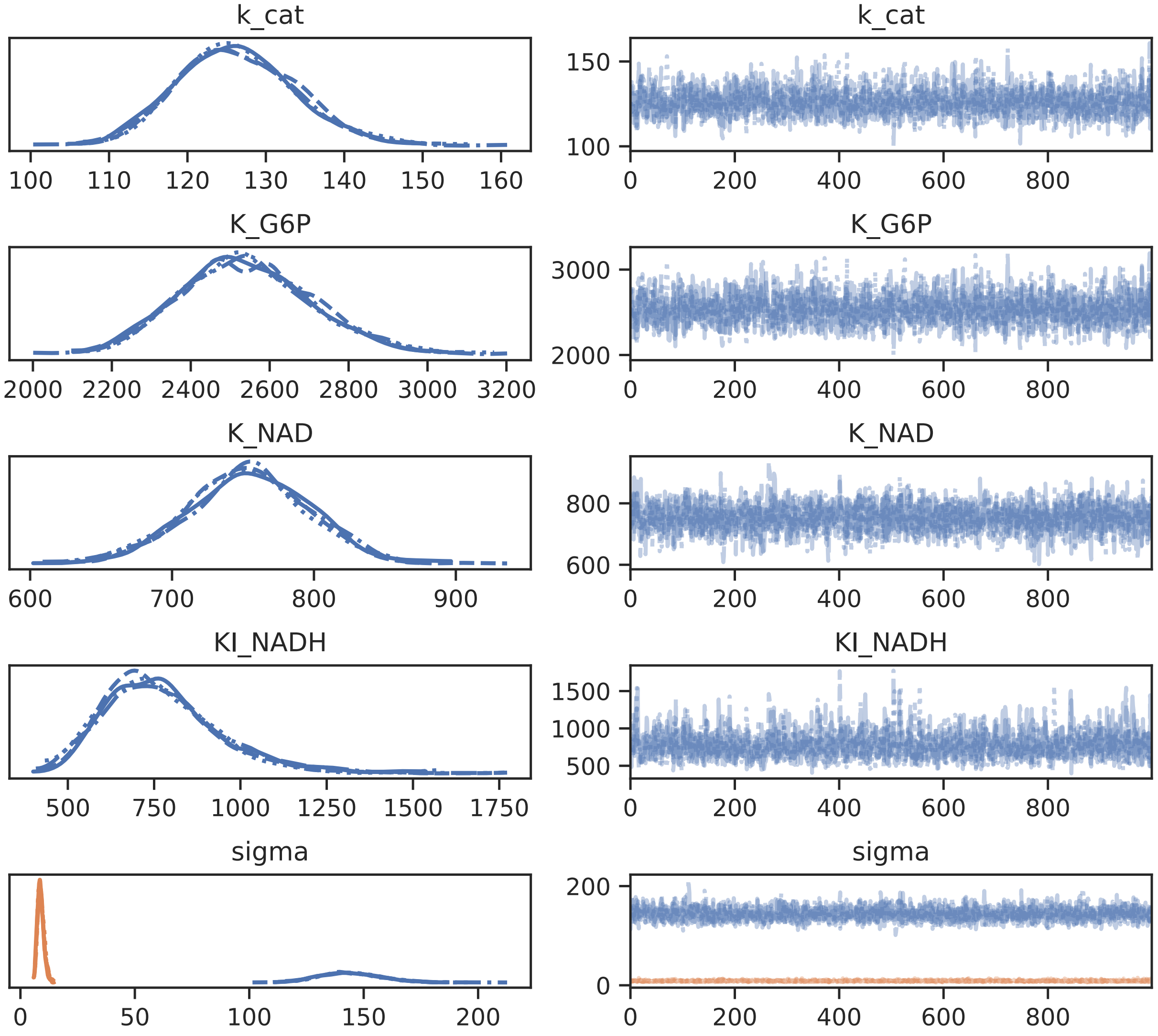

Figure S9: Diagnostic sampling trace plots of kinetic parameters inferred in manuscript Fig. 5, Hypothesis 1

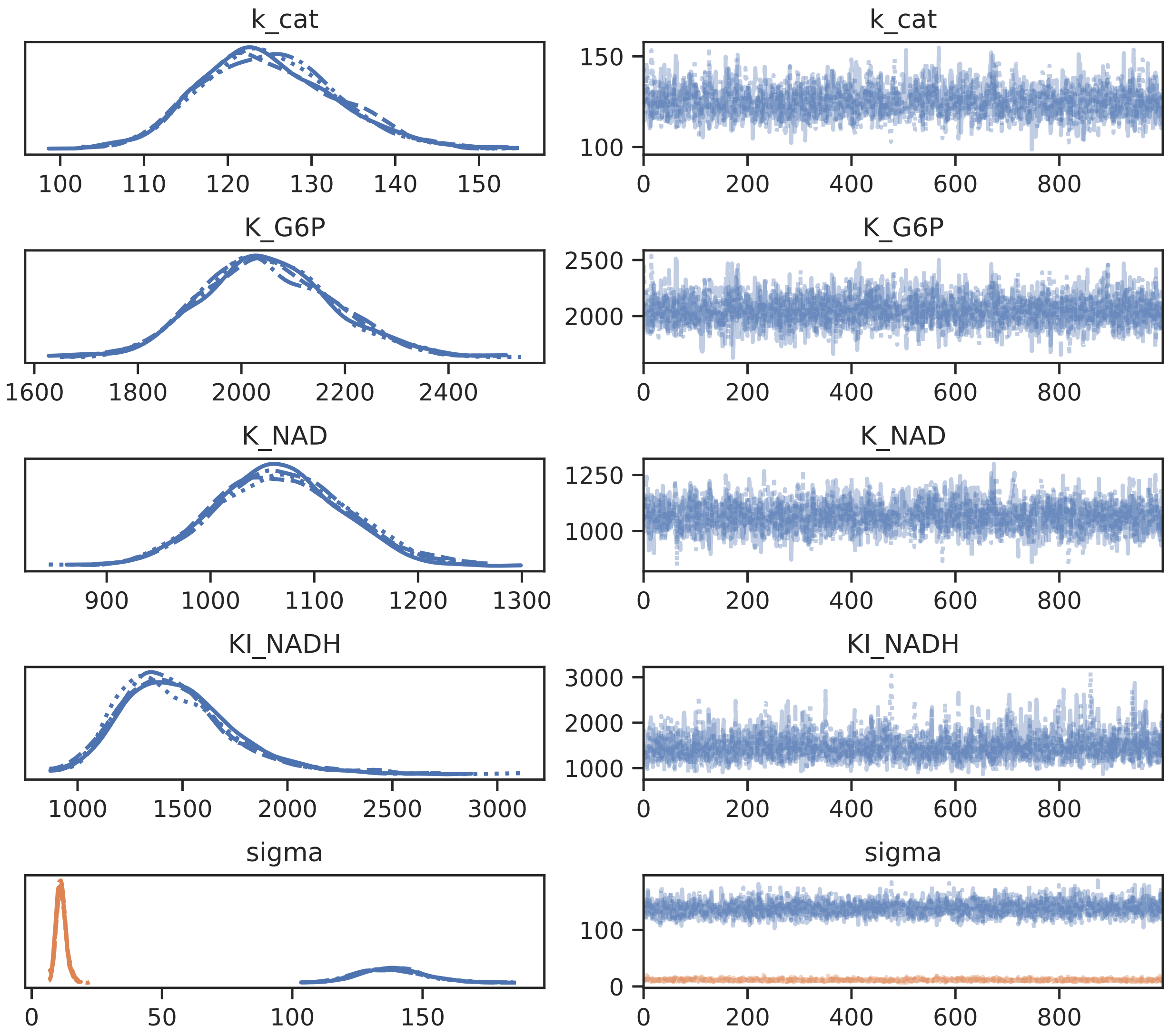

Figure S10: Diagnostic sampling trace plots of kinetic parameters inferred in manuscript Fig. 5, Hypothesis 2

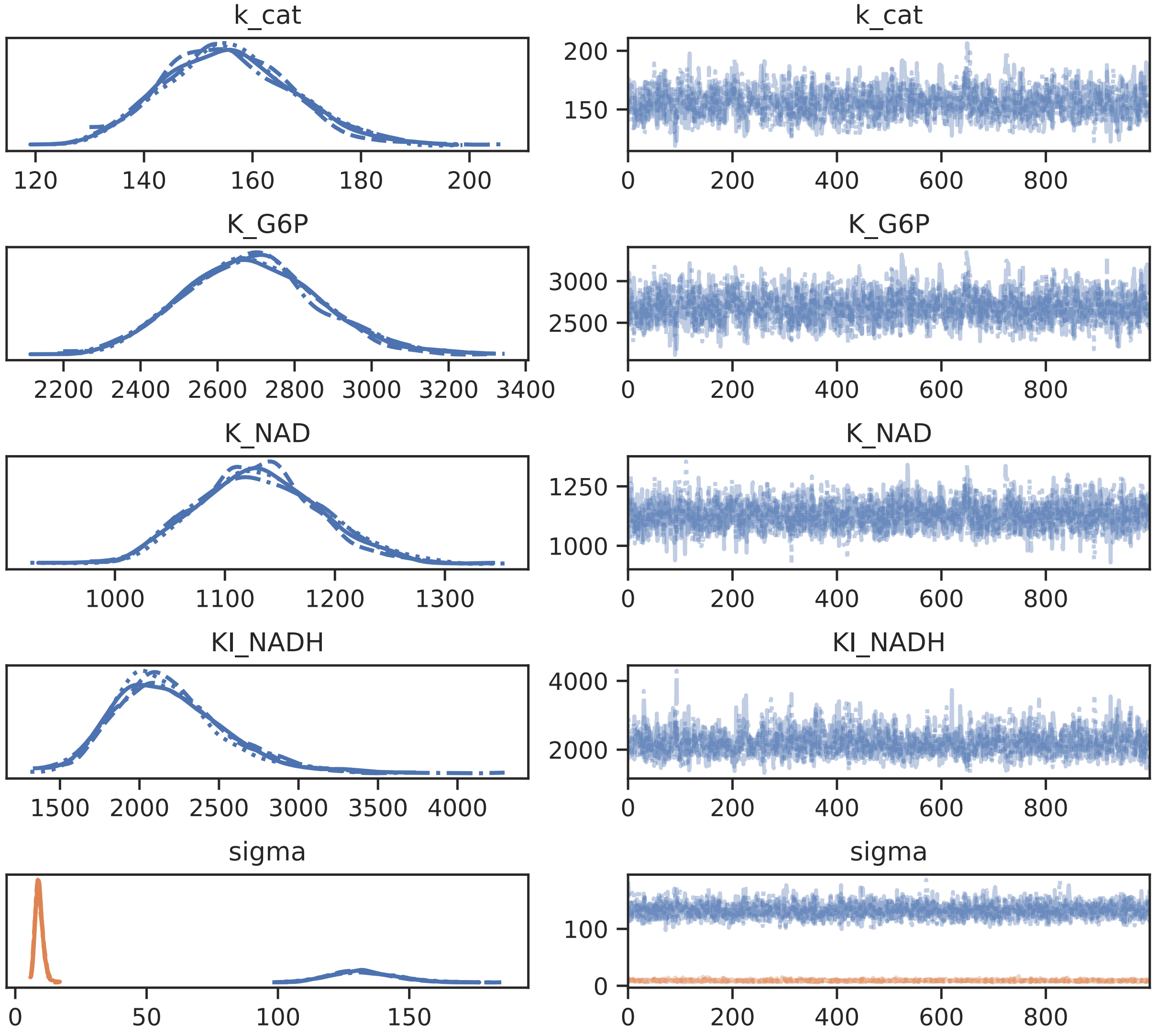

Figure S11: Diagnostic sampling trace plots of kinetic parameters inferred in manuscript Fig. 5, Hypothesis 3

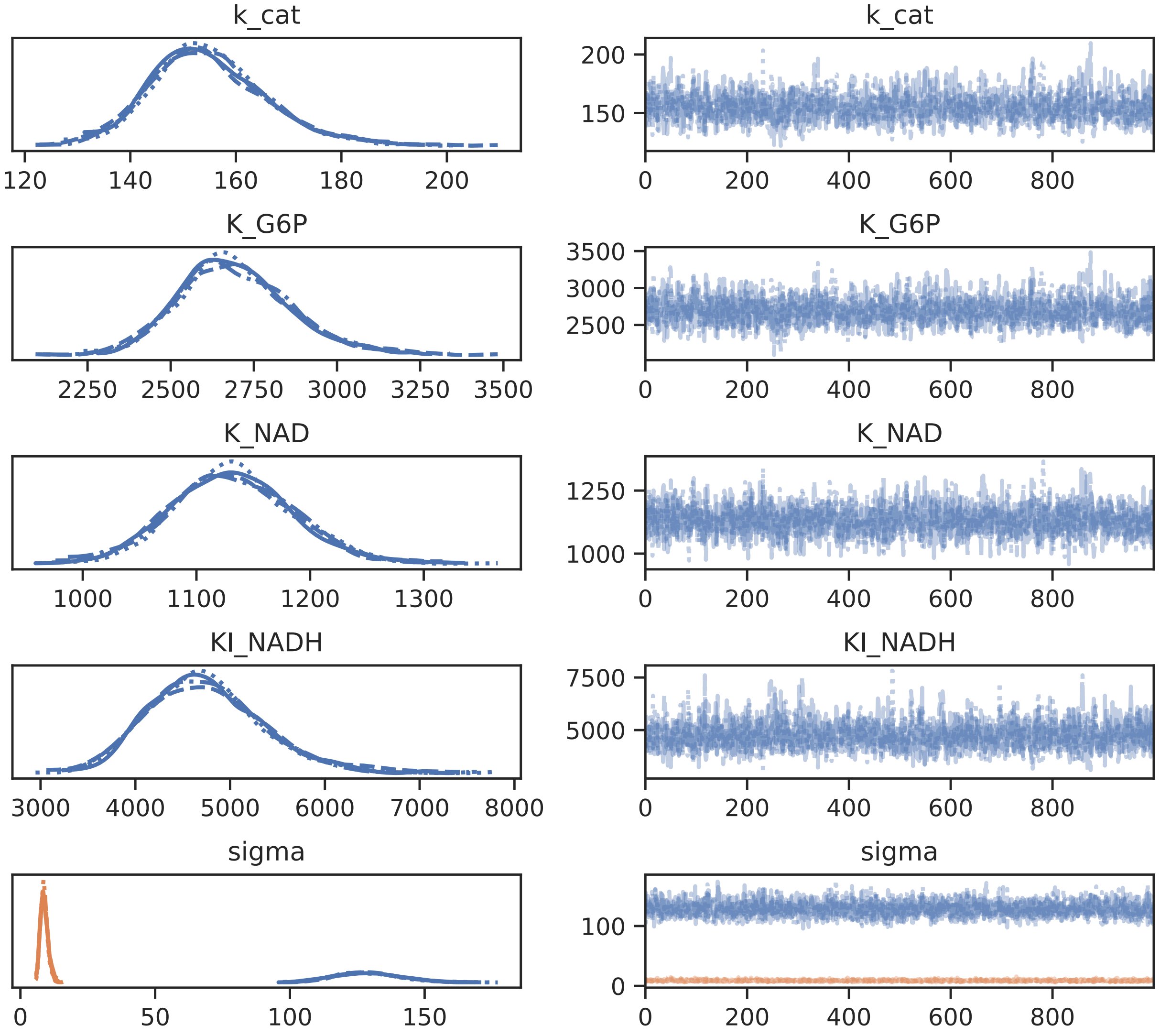

Figure S12: Diagnostic sampling trace plots of kinetic parameters inferred in manuscript Fig. 5, Hypothesis 4

References

(1) Klein, A. M.; Mazutis, L.; Akartuna, I.; Tallapragada, N.; Veres, A.; Li, V.; Peshkin, L.; Weitz, D. A.; Kirschner, M. W. Droplet Barcoding for Single-Cell Transcriptomics Applied to Embryonic Stem Cells. *Cell* **2015**, *161* (5), 1187–1201. https://doi.org/10.1016/j.cell.2015.04.044.

(2) Matula, K.; Rivello, F.; Huck, W. T. S. Single-Cell Analysis Using Droplet Microfluidics. *Advanced Biosystems* **2020**, *4* (1), 1900188. https://doi.org/10.1002/adbi.201900188.

(3) Semenov, S. N.; Markvoort, A. J.; Gevers, W. B. L.; Piruska, A.; Greef, T. F. A. de; Huck, W. T. S. Ultrasensitivity by Molecular Titration in Spatially Propagating Enzymatic Reactions. *Biophysical Journal* **2013**, *105* (4), 1057–1066. https://doi.org/10.1016/j.bpj.2013.07.002.

(4) Salvatier, J.; Wiecki, T. V.; Fonnesbeck, C. Probabilistic Programming in Python Using PyMC3. *PeerJ Computer Science* **2016**, *2*, e55. https://doi.org/10.7717/peerj-cs.55.

(5) Hoffman, M. D.; Gelman, A. The No-U-Turn Sampler: Adaptively Setting Path Lengths in Hamiltonian Monte Carlo. *arXiv:1111.4246 [cs, stat]* **2011**.

(6) Virtanen, P.; Gommers, R.; Oliphant, T. E.; Haberland, M.; Reddy, T.; Cournapeau, D.; Burovski, E.; Peterson, P.; Weckesser, W.; Bright, J.; Walt, S. J. van der; Brett, M.; Wilson, J.; Millman, K. J.; Mayorov, N.; Nelson, A. R. J.; Jones, E.; Kern, R.; Larson, E.; Carey, C. J.; Polat, İ.; Feng, Y.; Moore, E. W.; VanderPlas, J.; Laxalde, D.; Perktold, J.; Cimrman, R.; Henriksen, I.; Quintero, E. A.; Harris, C. R.; Archibald, A. M.; Ribeiro, A. H.; Pedregosa, F.; Mulbregt, P. van; Vijaykumar, A.; Bardelli, A. P.; Rothberg, A.; Hilboll, A.; Kloeckner, A.; Scopatz, A.; Lee, A.; Rokem, A.; Woods, C. N.; Fulton, C.; Masson, C.; Häggström, C.; Fitzgerald, C.; Nicholson, D. A.; Hagen, D. R.; Pasechnik, D. V.; Olivetti, E.; Martin, E.; Wieser, E.; Silva, F.; Lenders, F.; Wilhelm, F.; Young, G.; Price, G. A.; Ingold, G.-L.; Allen, G. E.; Lee, G. R.; Audren, H.; Probst, I.; Dietrich, J. P.; Silterra, J.; Webber, J. T.; Slavič, J.; Nothman, J.; Buchner, J.; Kulick, J.; Schönberger, J. L.; Miranda Cardoso, J. V. de; Reimer, J.; Harrington, J.; Rodríguez, J. L. C.; Nunez-Iglesias, J.; Kuczynski, J.; Tritz, K.; Thoma, M.; Newville, M.; Kümmerer, M.; Bolingbroke, M.; Tartre, M.; Pak, M.; Smith, N. J.; Nowaczyk, N.; Shebanov, N.; Pavlyk, O.; Brodtkorb, P. A.; Lee, P.; McGibbon, R. T.; Feldbauer, R.; Lewis, S.; Tygier, S.; Sievert, S.; Vigna, S.; Peterson, S.; More, S.; Pudlik, T.; Oshima, T.; Pingel, T. J.; Robitaille, T. P.; Spura, T.; Jones, T. R.; Cera, T.; Leslie, T.; Zito, T.; Krauss, T.; Upadhyay, U.; Halchenko, Y. O.; Vázquez-Baeza, Y. SciPy 1.0: Fundamental Algorithms for Scientific Computing in Python. **2020**, *17* (3), 261–272. https://doi.org/10.1038/s41592-019-0686-2.
